# A Coralline Algal-Associated Bacterium, *Pseudoalteromonas* Strain J010, Yields Five New Korormicins and a Bromopyrrole

**DOI:** 10.3390/md12052802

**Published:** 2014-05-13

**Authors:** Jan Tebben, Cherie Motti, Dianne Tapiolas, Peter Thomas-Hall, Tilmann Harder

**Affiliations:** 1 School of Biological, Earth and Environmental Sciences and Centre for Marine Bio-Innovation, The University of New South Wales, Sydney, NSW 2052, Australia; E-Mails: jan.tebben@unsw.edu.au (J.T.); t.harder@unsw.edu.au (T.H.); 2 Australian Institute of Marine Science, PMB no. 3, Townsville MC, Townsville 4810, Australia; E-Mails: d.tapiolas@aims.gov.au (D.T.); p.thomashall@aims.gov.au (P.T.-H.)

**Keywords:** *Pseudoalteromonas*, korormicin, bromopyrrole, bromoalterochromide, antibacterial, antifungal, antiprotozoal

## Abstract

The ethanol extract of *Pseudoalteromonas* strain J010, isolated from the surface of the crustose coralline alga *Neogoniolithon fosliei*, yielded thirteen natural products. These included a new bromopyrrole, 4′-((3,4,5-tribromo-1*H*-pyrrol-2-yl)methyl)phenol (**1**) and five new korormicins G–K (**2**–**6**). Also isolated was the known inducer of coral larval metamorphosis, tetrabromopyrrole (TBP), five known korormicins (A–E, previously named 1, 1a–c and 3) and bromoalterochromide A (BAC-A). Structures of the new compounds were elucidated through interpretation of spectra obtained after extensive NMR and MS investigations and comparison with literature values. The antibacterial, antifungal and antiprotozoal potential of **1**–**6**, TBP and BAC-A was assessed. Compounds **1**–**6** showed antibacterial activity while BAC-A exhibited antiprotozoal properties against *Tetrahymena pyriformis*. TBP was found to have broad-spectrum activity against all bacteria, the protozoan and the fungus *Candida albicans*.

## 1. Introduction

In the marine environment chemical signals play critical ecological roles at many organizational levels [1]. Epibiotic bacteria (e.g., associated with animals, plants, algae) are often the source of these signals [2].

In a previous bioassay-guided study of coral larval settlement cues [3], the pigmented bacterium *Pseudoalteromonas* strain J010, isolated from the surface of the crustose coralline alga (CCA) *Neogoniolithon fosliei*, triggered larval metamorphosis in acroporid corals [3]. The causative bacterial metabolite for the transition of planula larvae into non-attached primary polyps was identified as tetrabromopyrrole (TBP) [3]. During the course of this investigation other potentially new bacterial metabolites were encountered, including polybrominated pyrrole derivatives and a family of korormicin derivatives. Antimicrobial metabolites belonging to these compound classes have been previously isolated from other *Pseudoalteromonads* or closely related species [4,5,6]. Pigmented bacteria affiliated with the genus *Pseudoalteromonas* (class *Gammaproteobacteria*) have gained significant attention during the past decade as producers of a wide range of bioactive compounds [7].

In this study chemical analysis of *Pseudoalteromonas* strain J010 was undertaken and the new metabolites identified to better understand the metabolic capacity and antibiotic potential of this epiphytic bacterium. This study was motivated by the general notion that chemically undefended algae may benefit from bioactive secondary metabolites produced by associated bacteria [8], for example against bacterial colonization and fouling [9,10].

Details of the isolation and structural elucidation of the new bromopyrrole, 4′-((3,4,5-tribromo-1*H*-pyrrol-2-yl)methyl)phenol (**1**) and five new korormicins G–K (**2**–**6**) along with the known coral larval metamorphosis cue, TBP [3,4], five known korormicins (A–E, previously named 1, 1a–c and 3) [5,11,12] and bromoalterochromide A (BAC-A) [13] are provided. Their antimicrobial, antifungal and antiprotozoal activities are also described.

## 2. Results and Discussion

### 2.1. Isolation and Structural Elucidation of Bacterial Metabolites

NMR data and a characteristic MS isotopic distribution (3:10:10:3) established the molecular formula of compound **1** to be C_11_H_8_ONBr_3_, requiring seven degrees of unsaturation. The IR spectrum indicated the presence of both hydroxy (3388 cm^−1^) and amine (3271, 2925 cm^−1^) groups. Multiplicity-edited adiabatic HSQC data (Table 1) revealed a disubstituted double bond (δ_C_ 129.9, CH, C-8; δ_H_ 7.05, d, 8.3, H-8; 115.8, CH, C-9; δ_H_ 6.81, d, 8.3, H-9) and an isolated methylene (δ_C_ 32.5, CH_2_, C-6; δ_H_ 3.87, s, H-6). Furthermore, each proton signal in the ^1^H NMR integrated for two protons establishing the presence of two identical disubstituted double bonds and hence symmetry within the molecule. This together with HMBC correlations from H-8 to C-10 and from H-9 to δ_C_ 128.7 (C-7) as well as a *J*_8–9_ coupling of 8.3 Hz established a 1,4-disubstituted phenyl ring, accounting for four degrees of unsaturation in **1**. The position of the hydroxy moiety on the phenyl ring was determined by the downfield resonance at δ_C_ 155.1 (C-10) while a HMBC correlation from H8/12 to δ_C_ 32.5 (C-6) established the methylene side chain to be *para* substituted.

**Table 1:** NMR data (600 MHz and 125 MHz in CDCl_3_, Supplementary Table S1 and Figures S1–S14) for 4′-((3,4,5-tribromo-1*H*-pyrrol-2-yl)methyl)phenol (**1**).

Position	δ_C_, mult.	δ_H_ (*J* Hz)	gCOSY	gHMBC ^a^
NH	-	-	-	-
2	131.1, qC	-	-	-
3	97.8, qC	-	-	-
4	100.0, qC	-	-	-
5	100.0, qC	-	-	-
6	32.5, CH_2_	3.87, s	-	2, 3, 7, 8, 12
7	128.7, qC	-	-	-
8	129.9, CH	7.05, d (8.3)	9	2 ^b^, 6, 7, 9, 10, 11, 12
9	115.8, CH	6.81, d (8.3)	8	8, 10, 11, 12
10	155.1, qC	-	-	-
OH	-	-	-	-
11	115.8, CH	6.81, d (8.3)	12	8, 10, 11, 12
12	129.9, CH	7.05, d (8.3)	11	6, 8, 9, 10, 11

^a^ (^n^*J*_CH_ = 7.5 Hz), ^b^ (^n^*J*_CH_ = 12 Hz).

**Figure 1:**
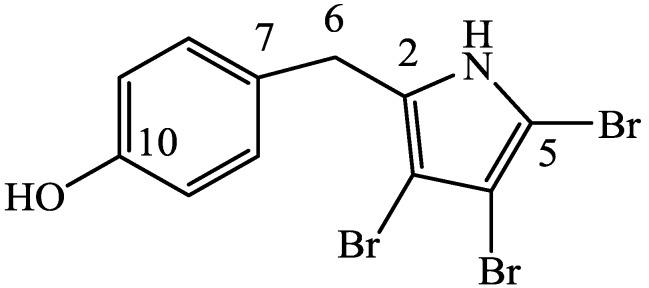
Structure of 4′-((3,4,5-tribromo-1*H*-pyrrol-2-yl)methyl)phenol (**1**).

The downfield resonance of C-2 (δ_C_ 131.1), HMBC correlations from H-6 to δ_C_ 131.1 (C-2) and δ_C_ 97.8 (C-3), plus the requirement for three bromines, established a tetrasubstituted pyrrole ring accounting for all seven degrees of unsaturation. Comparison of the carbon shifts of **1** with those of the natural and synthetic analogues 2,3,4-tribromopyrrole [14], 2,3,5-tribromopyrrole [14], pentabromopseudilin [15] and iso-pentabromopseudilin [15], as well as the reported expontaneous decomposition of iso-pentabromopseudilin [15], indicated that the two subunits were likely linked at C-2. This was supported by a weak HMBC correlation from H-8 to C-2 (Table 1 and Supplementary Figure S10). The structure of **1**, 4′-((3,4,5-tribromo-1*H*-pyrrol-2-yl)methyl)phenol, is as shown (Figure 1).

The molecular formula of compound **2** was established to be C_25_H_41_O_5_N from NMR and FTMS data. The presence of alcohol (3345 br cm^−1^), ester (1734 cm^−1^) and amide (1639 str, 1553 cm^−1^) functionality was determined by IR. In addition, NMR data (Table 2 and Table 3; Supplementary Figures S15–S18) for **2** was consistent with two conjugated, disubstituted double bonds (δ_C_ 129.3, CH, C-4′; 130.0, CH, C-5′; δ_H_ 5.39, dd, 11.3, 9.4, H-4′; δ_H_ 6.07, br t, 11.3, H-5′ and 126.4, CH, C-6′; 132.0, CH, C-7′; δ_H_ 6.47, br dd, 15.0, 11.3, H-6′; δ_H_ 5.82, br dt, 15.0, 6.8, H-7′) and two carbonyls (δ_C_ 174.7, C, C-2 and δ_C_ 172.0, C, C-1′), accounting for four of the six degrees of unsaturation; hence **2** is bicyclic. Also present (Table 2 and Table 3) was one quaternary carbon (δ_C_ 85.8, C, C-5), one C-N (δ_C_ 50.0, CH, C-3; δ_H_ 4.72, ddd, 11.5, 9.2, 5.6 Hz), three oxymethines (δ_C_ 64.7, CH; δ_H_ 5.01, br ddd, 9.4, 8.7, 3.2; δ_C_ 55.3, CH; δ_H_ 3.01, dt, 6.2, 4.2; δ_C_ 56.6, CH; δ_H_ 2.97, dt, 5.9, 4.2), eleven methylenes and three methyl groups.

**Table 2:** ^13^C NMR shifts of korormicins G–K (**2**–**6**).

No.	Korormicin					
	G (2)	H (3)	I (4)	I (4)	J (5)	K (6)
	δ_C_, mult. ^a^	δ_C_, mult. ^b^	δ_C_, mult. ^b^	δ_C_, mult. ^a^	δ_C_, mult. ^a^	δ_C_, mult. ^a^
1	-	-	-	-	-	-
2	174.7, qC	168.5, qC	168.5, qC	168.5, qC	nd ^c^	174.3, qC
3	50.0, CH	125.0, qC	125.0, qC	125.0, qC	50.0, CH	50.4, CH
4	39.9, CH_2_	133.7, CH	134.0, CH	134.0, CH	40.0, CH_2_	40.4, CH_2_
5	85.8, qC	87.2, qC	87.2, qC	87.2, qC	nd ^c^	85.6, qC
6	32.4, CH_2_	31.2, CH_2_	31.1, CH_2_	34.6, CH_2_	32.9, CH_2_	32.8, CH_2_
7	8.2, CH_3_	8.0, CH_3_	8.0, CH_3_	8.1, CH_3_	8.3, CH_3_	8.4, CH_3_
8	25.9, CH_3_	23.9, CH_3_	24.1, CH_3_	24.2, CH_3_	25.9, CH_3_	26.2, CH_3_
NH	-	-	-	-	-	-
1′	172.0, qC	170.1, qC	170.2, qC	170.2, qC	nd ^c^	171.7, qC
2′	42.3, CH_2_	43.9, CH_2_	44.0, CH_2_	43.1, CH_2_	42.7, CH_2_	42.7, CH_2_
3′	64.7, CH	63.5, CH	63.8, CH	64.6, CH	64.8, CH	65.0, CH
OH	-	-	-	-	-	-
4′	129.3 CH	132.8 CH	132.2 CH	129.4, CH	127.6, CH	129.0 CH
5′	130.0, CH	128.2, CH	128.5, CH	130.7, CH	130.6, CH	131.0, CH
6′	126.4, CH	127.8, CH	127.4, CH	127.4, CH	124.0, CH	124.7, CH
7′	132.0, CH	131.9, CH	132.5, CH	132.7, CH	136.6, CH	136.4, CH
8′	31.1, CH_2_	37.2, CH_2_	37.1, CH_2_	38.2, CH_2_	30.5, CH_2_	30.6, CH_2_
9′	55.3, CH	67.0, CH	72.1, CH	73.1, CH	128.3, CH	126.1, CH
OH	-	-	-	-	-	-
10′	56.6, CH	71.4, CH	67.7, CH	68.0, CH	129.2 CH	131.4, CH
11′	27.3, CH_2_	33.3, CH_2_	33.7, CH_2_	31.9, CH_2_	27.0, CH_2_	27.2, CH_2_
12′	29.1 ^d^, CH_2_	25.4, CH_2_	26.3, CH_2_	26.0, CH_2_	25.4, CH_2_	29.1 ^d^, CH_2_
13′	29.1 ^d^, CH_2_	29.0 ^d^, CH_2_	28.8 ^d^, CH_2_	28.8 ^d^, CH_2_	29.0 ^d^, CH_2_	29.1 ^d^, CH_2_
14′	29.1 ^d^, CH_2_	29.0 ^d^, CH_2_	28.6 ^d^, CH_2_	28.58 ^d^, CH_2_	28.6 ^d^, CH_2_	29.1 ^d^, CH_2_
15′	29.1 ^d^, CH_2_	29.0, CH_2_	28.6 ^d^, CH_2_	28.55 ^d^, CH_2_	31.4, CH_2_	29.1 ^d^, CH_2_
16′	31.6, CH_2_	31.3, CH_2_	31.2, CH_2_	31.7, CH_2_	22.0, CH_2_	31.6, CH_2_
17′	22.6, CH_2_	22.1, CH_2_	22.1, CH_2_	22.6, CH_2_	13.5, CH_3_	22.6, CH_2_
18′	13.9, CH_3_	13.9, CH_3_	13.9, CH_3_	13.9, CH_3_	-	14.1, CH_3_

^a^ CDCl_3_; ^b^ DMSO-*d*_6_; ^c^ No HMBC data was recorded for the qC due to low yield of **5**, these carbons remain unassigned; ^d^ interchangeable.

The COSY spectrum of **2** revealed three spin systems. The first, from H-3 (δ_H_ 4.72, ddd, 11.5, 9.2, 5.6) to NH (δ_H_ 6.58, d, 5.6) and H_a_-4 (δ_H_ 2.78, dd, 12.8, 9.2), and the second from H_2_-6 (δ_H_ 1.76, dq, 14.7, 7.5; 1.68, dq, 14.7, 7.5) to H_3_-7 (δ_H_ 1.0, t, 7.5), along with HMBC correlations from H_a_-4 to C-2 and C-3, and from H_3_-8 to C-4, C-5 and C-6 confirmed the presence of a 3,5,5-trisubstituted γ-lactone ring. The third spin system extended from H_2_-2′ to H_3_-18′. Coupling constants of *J*_4′–5′_ 11.3 Hz and *J*_6′–7′_ 14.5 Hz confirmed the *Z*- and *E*-configurations for each double bond, respectively. Further HMBC correlations from H_2_-2′ to C-1′ and C-3′ located this side chain at the amide carbonyl C-1′ attached to the γ-lactone ring, reminiscent of korormicin A [5,12].

**Table 3:** ^1^H NMR shifts of korormicins G–K (**2**–**6**).

No.	Korormicin					
	G (2)	H (3)	I (4)	I (4)	J (5)	K (6)
	δ_H_ (*J* Hz) ^a^	δ_H_ (*J* Hz) ^b^	δ_H_ (*J* Hz) ^b^	δ_H_ (*J* Hz) ^a^	δ_H_ (*J* Hz) ^a^	δ_H_ (*J* Hz) ^a^
1	-	-	-	-	-	-
2	-	-	-	-	-	-
3	4.72, ddd (11.5, 9.2, 5.6)	-	-	-	4.70, ddd (11.2, 9.2, 6.1)	4.70, ddd (11.2, 9.2, 5.6)
4	2.78, dd (12.8, 9.2) 1.90, dd (12.8, 11.5)	7.39, s	7.39, s	7.37, s	2.78, dd (12.8, 9.2) 1.90, dd (12.8, 11.2)	2.78, dd (12.9, 9.2) 1.90, dd (12.9, 11.2)
5	-	-	-	-	-	-
6	1.76, dq (14.7, 7.5) 1.68, dq (14.7, 7.5)	1.76, q (7.5)	1.76, q (7.4)	1.82, q (7.4)	1.77, dq (14.4, 7.5) 1.70, dq (14.4, 7.5)	1.76, dq (14.7, 7.5) 1.69, dq (14.7, 7.5)
7	1.0, t (7.5)	0.76, t (7.5)	0.76, t (7.4)	0.90, t (7.4)	1.00, t (7.5)	1.00, t (7.5)
8	1.47, s	1.41, s	1.41, s	1.50, s	1.49, s	1.47, s
NH	6.58, d (5.6)	9.91, br s	9.88, br s	8.22, br s	6.59, d (6.1)	6.60, d (5.6)
1′	-	-	-	-	-	-
2′	2.51, dd (15.4, 8.7) 2.45, dd (15.4, 3.2)	2.60, dd (14.4, 8.0) 2.41, dd (14.4, 4.6)	2.60, dd (14.4, 8.0) 2.41, dd (14.4, 5.2)	2.63, dd (15.8, 8.7) 2.58, dd (15.8, 3.1)	2.49, dd (15.5, 8.6) 2.45, dd (15.5, 3.2)	2.49, dd (15.3, 8.5) 2.45, dd (15.3, 3.5)
3′	5.01, br ddd (9.4, 8.7, 3.2)	4.85, br dddd (9.2, 8.0, 4.6, 4.1)	4.84, dddd (9.1, 8.0, 5.2, 4.7)	5.06, ddt (9.4, 8.7, 3.1)	5.01, m	5.00, ddd (9.4, 8.5, 3.5)
OH		5.16, br d (4.1)	5.11, d (4.7)			
4′	5.39, dd (11.3, 9.4)	5.30, br dd (11.1, 9.2)	5.28, dd (11.1, 9.1)	5.41, dd (11.1, 9.4)	5.35, dd (11.3, 9.4)	5.33, br dd (11.3, 9.4)
5′	6.07, br t (11.3)	5.92, t (11.1)	5.91, t (11.1)	6.09, t (11.1)	6.05, br t (11.3)	6.05, br t (11.3)
6′	6.47, br dd (15.0, 11.3)	6.45, br dd (14.8, 11.1)	6.41, br dd (14.8, 11.1)	6.45, br dd (14.9, 11.1)	6.34, br dd (14.9, 11.3,1.5)	6.34, ddd (14.7, 11.3, 0.8)
7′	5.82, br dt (15.0, 6.8)	5.71, br dt (14.8, 7.1)	5.71, br dt (14.8, 7.5, 7.2)	5.81, dt (14.9, 7.2)	5.75, dt (14.9, 6.7)	5.75, br dt (14.7, 6.9)
8′	2.36, br dd (6.8, 6.2)	2.62, m 2.45, br dd (15.7, 8.3)	2.32, br ddd (14.2, 7.2, 6.8) 2.28, br ddd (14.2, 7.5, 6.5)	2.44, br dd (7.2, 6.8)	2.87, br ddd (7.5, 6.7, 1.5)	2.87, ddd (7.3, 6.9, 1.0)
9′	3.01, dt (6.2, 4.2)	3.95, br ddd (8.3, 4.4, 3.1)	3.60, dddd (6.8, 6.5, 6.1, 3.1)	3.72, ddd (7.2, 6.8, 3.5)	5.37, ddd (10.9, 7.5, 1.5)	5.38, ddd (10.4, 7.3, 0.8)
9′-OH	-	-	5.01, br d (6.1)	-	-	-
10′	2.97, dt (5.9, 4.2)	3.56, m	3.91, ddd (9.8, 3.7, 3.1)	3.91, ddd (9.8, 3.9, 3.5)	5.47, ddd (10.9, 7.3, 1.5)	5.48, ddd (10.4, 7.3, 1.0)
10′-OH	-	4.95, br d (5.6)	-	-	-	-
11′	1.55, m	1.43, m	1.76, ddt (14.4, 7.1, 3.7) 1.66, ddt (14.1, 4.6, 9.8)	1.82, m 1.66, ddt (14.1, 4.6, 9.8)	2.04, m	2.05, dt (14.7, 7.3)
12′	1.44, m	1.26, m	1.45, m 1.33, m	1.43, m 1.33, m	1.39, m	1.36, m
13′	1.28–1.41	1.20–1.36	1.20–1.36	1.20–1.36	1.20–1.35	1.28–1.41
14′	1.28–1.41	1.20–1.36	1.20–1.36	1.20–1.36	1.20–1.35	1.28–1.41
15′	1.28–1.41	1.20–1.36	1.20–1.36	1.20–1.36	1.24, m	1.28–1.41
16′	1.30, m	1.25, m	1.24, m	1.26, m	1.32, m	1.30, m
17′	1.32, m	1.27, m	1.27, m	1.28, m	0.89, t (7.0)	1.30, m
18′	0.89, t (7.0)	0.86, t (6.9)	0.86, t (6.8)	0.88, t (7.2)	-	0.89, t (6.9)

^a^ CDCl3; ^b^ DMSO-*d*_6_.

**Figure 2:**
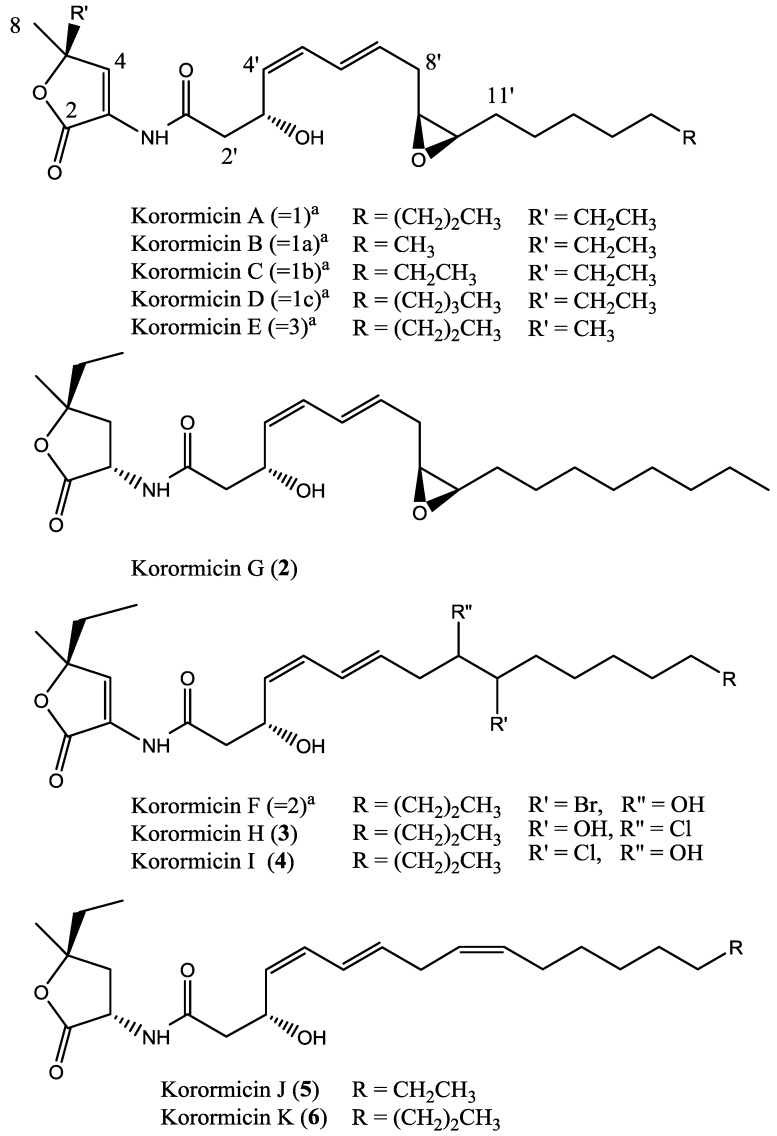
Structures of korormicins G–K (**2**–**6**). ^a^ nomenclature used by Yoshikawa *et al.* [11].

An epoxide was deduced at C-9′ and C-10′ based on the ^13^C chemical shifts (δ_C_ 55.3, CH; δ_C_ 56.6, CH), the COSY correlations from δ_H_ 3.01 (H-9′) to δ_H_ 2.97 (H-10′) and a C-O-C stretch at 1103 cm^−1^. As for korormicin A [5], the *J*_9′–10′_ vicinal coupling constant of 4.2 Hz confirmed the epoxide to be *cis*. The remaining hydroxy group was located at C-3′ (δ_C_ 64.7). Therefore, compound **2** (Figure 2) is the saturated γ-lactone analogue of korormicin A [5,12]. Yoshikawa *et al.* reported the isolation and structural elucidation of six korormicins (korormicin 1, 1a–c, 2 and 3) [5,11]. We propose that these korormicins be recognised for nomenclature purposes as korormicins A–F respectively, hence compound **2** is named korormicin G.

The molecular formula of **3** was established as C_25_H_40_O_5_NCl based on ^13^C NMR (Table 2) and FTMS data, requiring six degrees of unsaturation. Analysis of the ^1^H and ^13^C NMR data (Table 2 and Table 3; Supplementary Figures S19–S23) of **3** indicated it was similar to that of **2**, with two notable differences. Compound **3** contained a tri-substituted double bond (δ_C_ 125.0, C, C-3; 133.7, CH, C-4; δ_H_ 7.39, s, H-4) which, based on HMBC correlations from δ_H_ 7.39 to C-2 and C-5, confirmed it to have the same γ-lactone ring present in korormicin A [5]. In addition, the ^13^C chemical shifts of C-9′ (δ_C_ 67.0, CH) and C-10′ (δ_C_ 71.4, CH) along with the shift downfield of their respective protons (δ_H_ 3.95, br ddd, 8.3, 4.4, 3.1, H-9′; δ_H_ 3.56, m, H-10′) indicated the epoxide in **2** was no longer present in **3**. Moreover, a COSY correlation from δ_H_ 4.95 (br d, 5.6) to H-10′ confirmed the presence of a hydroxy moiety at C-10′ and hence the location of the chlorine at C-9′. Comparison of the 1D NMR data of **3** with korormicin F [11] confirmed the planar structure of **3**, korormicin H, is as shown in Figure 2.

As for **3**, the molecular formula of **4** was determined to be C_25_H_40_O_5_NCl. The ^13^C NMR data (Table 2; Supplementary Figures S24–S32) of **4** closely resembled that of **3**, except the ^13^C chemical shifts of C-9′ and C-10′ were reversed. COSY, HSQC and HMBC correlations, and comparison with literature values of korormicin F [11], confirmed the position of the chlorine at C-10′ and the planar structure of **4**, korormicin I, to be as shown in Figure 2. The configuration at C-9′ and C-10′ remains unresolved for both **3** and **4**.

Compound **5** was determined, by NMR and FTMS to have the molecular formula C_24_H_39_O_4_N. The NMR data (Table 2 and Table 3; Supplementary Figures S33–S35) of **5** showed strong similarity to **2**, with signals diagnostic of the amide substituted γ-lactone ring (δ_C_ 50.0, CH; δ_H_ 4.70, ddd, 11.2, 9.2, 6.1; δ_C_ 40.0, CH_2_; δ_H_ 2.78, dd, 12.8, 9.2, and 1.90, dd, 12.8, 11.2; δ_H_ 6.59, NH, d, 6.1). Downfield resonances indicating the presence of an isolated double bond (δ_C_ 128.3, CH; δ_H_ 5.37, dd, 10.9, 7.5, 1.5; δ_C_ 129.2, CH δ_H_ 5.47, ddd, 10.9, 7.3, 1.5; *cis* geometry) were observed in place of those for the epoxide in **2**. FTMS and ^13^C NMR data also confirmed that **5** had one less methlyene unit than **2**, hence the planar structure of **5**, korormicin J, is as shown (Figure 2). Several attempts to re-isolate **5** were futile; as a result the quaternary carbons remain unassigned (Table 2).

The final compound isolated in this study, compound **6**, was established as having the molecular formula C_25_H_41_O_4_N by FTMS. The NMR data (Table 2 and Table 3; Supplementary Figures S36–S39) for **6** were similar to that of **5**, the only difference being the addition of a methylene unit in the alkyl side chain. The configuration of the three double bonds in both compounds **5** and **6** was determined from their ^1^H NMR coupling constants, *J*_4′,5′_ (11.3 and 11.3), *J*_6′,7′_ (14.9 and 14.7), and J_9′,10′_ (10.9 and 10.4), to be *Z*, *E* and *Z*, respectively. The planar structure of **6**, korormicin K, is as shown in Figure 2.

The biosynthetic pathway of the korormicins has not yet been elucidated, however, this compound class shares structural similarity with acyl-homoserine lactones (AHL), which are also produced by *Pseudoalteromonas* spp. and whose biosynthesis has been described [16]. Given this, it is plausible that there would be some homology between the two biosynthetic pathways giving rise to compound classes which share the same configuration, especially at C-3 in the γ-lactone ring. Based on this assumption it is likely that compound **2** shares the same configurations at positions C-5, C-3′, C-9′ and C-10′ (5S*, 3′R*, 9′*S* *, 10′R*) with korormicin A. Likewise, positions C-5 and C-3′ for compounds **5** and **6** were assumed to be 5S* and 3′R*. For compounds **2**, **5** and **6** key nOe correlations from H-3 to H-6 and H-7 confirmed that in each case H-3 and the ethyl group were both on the same face of the molecule, hence **2**, **5** and **6** have the relative configuration 3S*, 5S*. The configurations at C-9′ and C-10′ remain unassigned for compounds **3** and **4**.

### 2.2. Antibacterial, Antifungal and Antiprotozoal Activities of Bacterial-Derived Metabolites

The activities of TBP, BAC-A and compounds **1**–**4**, **6** against twelve bacterial strains and one fungal strain are presented in Table 4. Due to the low yield, compound **5** was not tested.

TBP exhibited broad-spectrum activity against all bacterial strains tested and was also the only compound to display antifungal activity. Similarly, the bromopyrrole **1** showed broad-spectrum antibacterial activity; however, it did not inhibit fungal growth nor was it active against the source *Pseudoalteromonas* strain J010. BAC-A, on the other hand, did not show any antibacterial or antifungal activity. TBP and **1** were also the only compounds to exhibit activity against the gram-positive bacterium, *Staphylococcus aureus*. The antibacterial activity of the new korormicins in this study is in accordance with previous reports [5,11,17]. The korormicins **2**–**4** and **6** did not, however, inhibit the growth of either the gram-positive bacterium or the fungus, and similar to **1**, did not inhibit the CCA-derived *Pseudoalteromonas* strain J010.

These *in vitro* results imply an allelopathic role for *Pseudoalteromonas* strain J010-derived metabolites particularly against the pathogenic *Vibrios* indicating a putative role(s) in protecting the algal host. However, it remains to be shown if these metabolites evoke similar effects *in vivo*.

The antiprotozoal activity of TBP and BAC-A was 2.6 µM and 59.2 µM, respectively. At these concentrations all flagellates formed cysts within 5 min of incubation, with complete cell lysis within 30 min. These results raise the possibility that TBP- and BAC-A-producing bacteria may consequently be defended against protozoan grazing. None of the new compounds **1**–**4** and **6** exhibited antiprotozoal activity.

**Table 4:** Antibacterial, antifungal and antiprotozoal activities of bromoalterochromide A (BAC-A), tetrabromopyrrole (TBP), 4′-((3,4,5-tribromo-1*H*-pyrrol-2-yl)methyl)phenol (**1**) and korormicins G–I and K (**1**–**4**, **6**); bacterial and fungal inhibition was determined with 40 µg compound/diffusion disc. Growth media: LB10—LB10 agar, MA—Marine Agar, NSS—Nine Salts Solution.

Strains (Growth media; temperature °C)	Bromopyrroles			Korormicins G–I and K			
	BAC-A	TBP	1	2	3	4	6
*Pseudoalteromonas haloplanctis* (MA; 28)	n	y	y	y	y	y	y
*Pseudoalteromonas piscicida* (MA; 28)	n	y	y	y	y	y	y
*Pseudoalteromonas undina* (MA; 28)	n	y	y	y	y	y	y
*Pseudoalteromonas* strain. J010 (MA; 28)	n	y	n	n	n	n	n
*Pseudomonas aeruginosa* (LB10; 37)	n	y	y	y	y	y	y
*Vibrio campbellii* (MA; 28)	n	y	y	y	y	y	y
*Vibrio vulnificus* (LB10; 37)	n	y	y	y	y	y	y
*Vibrio coralliilyticus* (MA; 28)	n	y	y	y	y	y	y
*Vibrio harveyi* (MA; 28)	n	y	y	y	y	y	y
*Shewanella aquimarina* (MA; 28)	n	y	y	y	y	y	y
*Staphylococcus aureus* (LB10; 37)	n	y	y	n	n	n	n
*Candida albicans* (MA; 28)	n	y	n	n	n	n	n
*Tetrahymena pyriformis* (NSS; ambient)	y	y	n	n	n	n	n

## 3. Experimental Section

### 3.1. General Experimental Procedures

General experimental details and instrumentation have been previously reported [18]. Reversed-phase C_18_ material (Sepra C18-e, 50 µM) and HPLC columns were purchased from Phenomenex (Sydney, Australia). Solvents used were HPLC grade (Mallinckrodt, Sydney, Australia). All other chemicals were sourced from Sigma-Aldrich (Sydney, Australia). A Perkin Elmer Spectrum 100 FTIR spectrophotometer was used to record all IR spectra. ^1^H and ^13^C NMR spectra were recorded in either CDCl_3_ or DMSO-*d*_6_ (Cambridge Isotopes Laboratories Inc., Novachem, Melbourne, Australia) using 3 mm Bruker MATCH NMR tubes on a Bruker Avance 600 MHz NMR spectrometer with cryoprobe. Low-resolution MS data of the fractions were measured by direct infusion on a Bruker Daltonics Esquire 3000 ion-trap mass spectrometer (MS). Accurate mass for each compound was measured with a Bruker BioApex 47e FT-ICR mass spectrometer.

### 3.2. Isolation of Bacterial Metabolites

Petri dishes (*n* = 300, 2% marine agar) were inoculated with a stock culture of *Pseudoalteromonas* strain J010 and incubated for 48 h at 28 °C. Bacterial colonies were harvested with a sterile spatula, pooled and the bacterial biomass (25 g) extracted with ethanol (3 × 50 mL). The pooled ethanol extract was dried *in vacuo*, dissolved in 20 mL of methanol (MeOH), and purified on a pre-equilibrated C18 flash chromatography column (5 × 100 cm). The column was flushed with two bed volumes of MeOH and the eluant dried *in vacuo* then re-dissolved in 2 mL MeOH. The sample was separated on a Luna Phenyl-Hexyl column (250 × 21.2 mm, 5 µM) under gradient elution from 70:30 MeOH:H_2_O to 100% MeOH over 30 min, followed by 6 min with 100% MeOH with a flow rate of 8 mL/min. Fractions collected according to the peak profile at λ 220 nm. Low resolution MS data of the fractions was also measured by direct infusion. All individual compounds were further purified by C18 analytical HPLC (250 × 4.6 mm, 5µ Luna 2); 1 mL/min with gradient elution using H_2_O and acetonitrile. The chromatography yielded (in order of elution) BAC-A ([13]; rt 10.9 min, 5.2 mg, 0.02%); 4′-((3,4,5-tribromo-1*H*-pyrrol-2-yl)methyl)phenol (**1**; rt 19.25 min, 18.3 mg, 0.06%), korormicin B (previously 1a [11]; rt 20 min, 24.6 mg, 0.08%), TBP ([3,4]; rt 20.8 min, 26.2 mg, 0.10%), korormicin G (**2**; rt 21.3 min, 14.7 mg, 0.05%), korormicin E (previously 1c [11]; rt 21.6 min, 7.5 mg, 0.03%), korormicin H (**3**; rt 21.6 min, 7.9 mg, 0.03%), korormicin I (**4**; rt 21.7 min, 31.2 mg, 0.12%), korormicin A (previously 1 [5]; rt 24.6 min, 121.3 mg, 0.48%), korormicin C (previously 1b [11]; rt 23.8 min, 15.0 mg, 0.06%), korormicin J (**5**, rt 24.60 min, 1.0 mg, 0.004%), korormicin D (previously 3 [11]; rt 24.8 min, 26.4 mg, 0. 11%) and korormicin K (**6**; rt 25.38 min, 12.8 mg, 0.05%). The percentage yield of all compounds isolated was based on the weight of bacterial biomass. The known compounds had identical physical and spectroscopic properties to those previously published [3,4,5,11,13].

#### 3.2.1. 4′-((3,4,5-Tribromo-1H-pyrrol-2-yl)methyl)phenol (**1**)

Colourless oil. UV (PDA) λ_max_ nm: 229, 278; IR ν_max_ cm^−1^: 3388 br, 3271, 2925, 1655, 1024, 992, 825; ^1^H (600 MHz, CDCl_3_ and DMSO-*d*_6_) and ^13^C (125 MHz, CDCl_3_ and DMSO-*d*_6_) NMR data Table 1 and Supplementary Figures S1–S13; (−)-ESI-FTMS *m*/*z* 405.8100 (calcd for C_11_H_7_ONBr_3_^−^ monoisotopic 405.8083).

#### 3.2.2. (3S*, 5S*, 3′R*, 4′*Z*, 6′*E*, 9′S*, 10′R*) Korormicin G (**2**)

Colourless oil. [α]^21^_D_ not determined UV (PDA) λ_max_ nm: 232; IR ν_max_ cm^−1^: 3345, 2914, 1639 str, 1620, 1553, 1388, 1237, 1103, 1044; ^1^H (600 MHz, CDCl_3_) Table 3 and ^13^C (125 MHz, CDCl_3_) NMR data Table 2 and Supplementary Figures S14–S17; (+)-ESI-FTMS *m*/*z* 458.2863 (calcd for C_25_H_41_O_5_NNa^+^ 458.2877).

#### 3.2.3. (3S*, 3′R*, 4′*Z*, 6′*E*) Korormicin H (**3**)

Colourless oil. [α]^21^_D_ not determined UV (PDA) λ_max_ nm: 230; ^1^H (600 MHz, DMSO-*d*_6_) Table 3 and ^13^C (125 MHz, DMSO-*d*_6_) NMR data Table 2 and Supplementary Figures S18–S22; (+)-ESI-FTMS *m*/*z* 492.2497 (calcd for C_25_H_40_O_5_NClNa^+^ 492.2487).

#### 3.2.4. (3S*, 3′R*, 4′*Z*, 6′*E*) Korormicin I (**4**)

Colourless oil. [α]^21^_D_ not determined UV (PDA) λ_max_ nm: 232; ^1^H (600 MHz, DMSO-*d*_6_) Table 3 and ^13^C (125 MHz, DMSO-*d*_6_) NMR data Table 2 and Supplementary Figures S23–S31; (+)-ESI-FTMS *m*/*z* 492.2499 (calcd for C_25_H_40_O_5_NClNa^+^ 492.2487).

#### 3.2.5. (3S*, 5S*, 3′R*, 4′*Z*, 6′*E*, 9′Z*) Korormicin J (**5**)

Colourless oil. [α]^21^_D_ not determined UV (PDA) λ_max_ nm: 233; ^1^H (600 MHz, CDCl_3_) Table 3 and ^13^C (125 MHz, CDCl_3_) NMR data Table 2 and Supplementary Figure S32–S34; (+)-ESI-FTMS *m*/*z* 428.2768 (calcd for C_24_H_39_O_4_NNa^+^ 428.2771).

#### 3.2.6. (3S*, 5S*, 3′R*, 4′*Z*, 6′*E*, 9′Z*) Korormicin K (**6**)

Colourless oil. [α]^21^_D_ 17° (CH_3_OH; *c* 0.6); UV (PDA) λ_max_ nm: 232; ^1^H (600 MHz, CDCl_3_) Table 3 and ^13^C (125 MHz, CDCl_3_) NMR data Table 2 and Supplementary Figures S35–S38; (+)-ESI-FTMS *m*/*z* 442.2919 (calcd for C_25_H_41_O_4_NNa^+^ 442.2928).

### 3.3. Antibacterial, Antifungal and Antiprotozoal Bioassays

The disc diffusion assay was performed according to protocols described in Bauer *et al.* [19]. Briefly, fractions were applied without drying while pure compounds **1**, BAC-A and TBP were dissolved in MeOH and **2**–**6** in CHCl3 at 200 μg/mL. Aliquots (20 μL) of fractions and compounds were evaporated on filter paper discs (*d* = 6 mm, Bio-Rad, Sydney, Australia) and placed on petri dishes containing Marine Agar or LB agar depending on the target strain with solvent as the negative control. Each petri dish was inoculated with one of the following bacterial strains: the Gram positive *Staphylococcus aureus*, or the Gram negative bacterial strains: *Pseudoalteromonas* strain J010, *Pseudoalteromonas haloplanctis*, *Pseudoalteromonas piscicida*, *Pseudoalteromonas undina*, *Pseudomonas aeruginosa*, *Shewanella aquimarina*, *Vibrio campbellii*, *V. coralliilyticus*, *V. harveyi* and *V. vulnificus*. Fractions and compounds were also tested against the fungus *Candida albicans*. Petri dishes were incubated for 24 h (at the temperature given in Table 4) after which clearance zones >10 mm in diameter (2 mm zone around the disc) were deemed as being inhibited by the target compound.

An axenic culture of the ciliate *Tetrahymena pyriformis* was used to assess antiprotozoal effects according to Matz *et al.* [20]. Briefly, samples were dried under vacuum, suspended in EtOH and 1 to 200 μg/mL transferred into 24-well plates. Once dry, Nine Salts Solution medium [21] with 10^3^ flagellates per mL was added to each well (500 µL final volume per well) and total flagellate and active cell numbers monitored using an inverted light microscope.

## 4. Conclusions

Chemical screening of the epiphytic *Pseudoalteromonas* strain J010, isolated from the surface of the CCA *N. fosliei*, yielded six new compounds: the unprecedented bromopyrrole, 4′-((3,4,5-tribromo-1*H*-pyrrol-2-yl)methyl)phenol (**1**) and the five korormicins G–K (**2**–**6**), together with the seven known compounds: korormicins A–E [5,11], BAC-A [4] and TBP [3,4]. The metabolites identified in this study were shown to elicit antagonistic effects against bacteria, fungi, and protozoa, similar to previous reports [22,23,24,25]. Given the korormicins share a structural scaffold with the AHLs it is also possible that these compounds, like the long-chain (C_13–16_ and C_18_) AHLs, may play a role in quorum sensing [26]. Bromopyrroles have also previously been shown to have feeding deterrent [27,28] and antineoplastic properties [29].

Based on its metabolic capacity and antibiotic potential, it is tempting to speculate that the epiphytic bacterium *Pseudoalteromonas* strain J010 is highly defended against both eukaryotic and prokaryotic microbes in its ecological niche. This may have further implications for the macroscopic host. To test these hypotheses, both the bacterial abundance and the concentrations of bioactive metabolites need to be measured and shown to occur above active thresholds *in vivo*.

## Supplementary Files

Supplementary File 1 Supplementary Information (PDF, 3009 KB)
