# Supplementary material for: A Coralline Algal-Associated Bacterium, Pseudoalteromonas Strain J010, Yields Five New Korormicins and a Bromopyrrole
Source: Mar Drugs. 2014 May 13;12(5):2802–15. doi: 10.3390/md12052802 (PMC4052317; doi:10.3390/md12052802)
Supplement: Supplementary File 1 — Supplementary Information (PDF, 3009 KB) [file marinedrugs-12-02802-s001.pdf]

Supplementary Information

Figure S1. ^{1}H spectrum (600 MHz) of 4′-((3,4,5-tribromo-1*H*-pyrrol-2-yl)methyl)phenol (**1**) in DMSO-d_6.
Figure S2. COSY spectrum (600 MHz) of (**1**) in DMSO-d_6.
Figure S3. HSQC spectrum (600 MHz) of (**1**) in DMSO-d_6.
Figure S4. HMBC spectrum (600 MHz) of (**1**) in DMSO-d_6.
Figure S5. ^{1}H spectrum (600 MHz) of 4′-((3,4,5-tribromo-1*H*-pyrrol-2-yl)methyl)phenol (**1**) in CDCl3.
Figure S6. ^{13}C NMR spectrum (125 MHz) of (**1**) in $CDCl_3$.
Figure S7. COSY spectrum (600 MHz) of (**1**) in $CDCl_3$.
Figure S8. HSQC spectrum (600 MHz) of (**1**) in $CDCl_3$.
Figure S9. HMBC spectrum (600 MHz) of (**1**) in $CDCl_3$ J_{CH} = 7.5 Hz.
Figure S10. HMBC spectrum (600 MHz) of (**1**) in $CDCl_3$ J_{CH} = 12 Hz.
Figure S11. ^{1}H spectrum (600 MHz) of (**1**) in CD_3OD.
Figure S12. ^{13}C NMR spectrum (125 MHz) of (**1**) in CD_3OD.
Figure S13. HSQC spectrum (600 MHz) of (**1**) in CD_3OD.
Figure S14. HMBC spectrum (600 MHz) of (**1**) in CD_3OD.
Table S1. NMR data (600 MHz and 125 MHz, Figures S1–S14) for 4′-((3,4,5-tribromo-1*H*-pyrrol-2-yl)methyl)phenol (**1**).
Figure S15. ^{1}H spectrum (600 MHz) of Korormicin G (**2**) in $CDCl_3$.
Figure S16. COSY spectrum (600 MHz) of (**2**) in $CDCl_3$.
Figure S17. HSQC spectrum (600 MHz) of (**2**) in $CDCl_3$.
Figure S18. HMBC spectrum (600 MHz) of (**2**) in $CDCl_3$.
Figure S19. ^{1}H spectrum (600 MHz) of Korormicin H (**3**) in DMSO-d_6.
Figure S20. ^{13}C NMR spectrum (125 MHz) of (**2**) in DMSO-d_6.
Figure S21. COSY spectrum (600 MHz) of (**3**) in DMSO-d_6.
Figure S22. HSQC spectrum (600 MHz) of (**3**) in DMSO-d_6.
Figure S23. HMBC spectrum (600 MHz) of (**3**) in DMSO-d_6.
Figure S24. ^{1}H spectrum (600 MHz) of Korormicin I (**4**) in $CDCl_3$.
Figure S25. COSY spectrum (600 MHz) of (**4**) in $CDCl_3$.
Figure S26. HSQC spectrum (600 MHz) of (**4**) in $CDCl_3$.
Figure S27. ^{1}H spectrum (600 MHz) of Korormicin I (**4**) in DMSO-d_6.
Figure S28. ^{13}C NMR spectrum (125 MHz) of (**4**) in DMSO-d_6.
Figure S29. COSY spectrum (600 MHz) of (**4**) in DMSO-d_6.
Figure S30. HSQC spectrum (600 MHz) of (**4**) in DMSO-d_6.
Figure S31. HMBC spectrum (600 MHz; cnst13=12) of (**4**) in DMSO-d_6.
Figure S32. HMBC spectrum (600 MHz; cnst13=7.5) of (**4**) in DMSO-d_6.
Figure S33. ^{1}H spectrum (600 MHz) of Korormicin J (**5**) in $CDCl_3$.
Figure S34. COSY spectrum (600 MHz) of (**5**) in $CDCl_3$.
Figure S35. HSQC spectrum (600 MHz) of (**5**) in $CDCl_3$.
Figure S36. ^{1}H spectrum (600 MHz) of Korormicin K (**6**) in $CDCl_3$.
Figure S37. COSY spectrum (600 MHz) of (**6**) in $CDCl_3$.
Figure S38. HSQC spectrum (600 MHz) of (**6**) in $CDCl_3$.
Figure S39. HMBC spectrum (600 MHz) of (**6**) in $CDCl_3$.
Figure S40. Selective nOe spectrum (600 MHz) of (**6**); H-3 in CDCl3.

Figure S1. ^{1}H spectrum (600 MHz) of 4′-((3,4,5-tribromo-1*H*-pyrrol-2-yl)methyl)phenol (**1**) in DMSO-d_6.

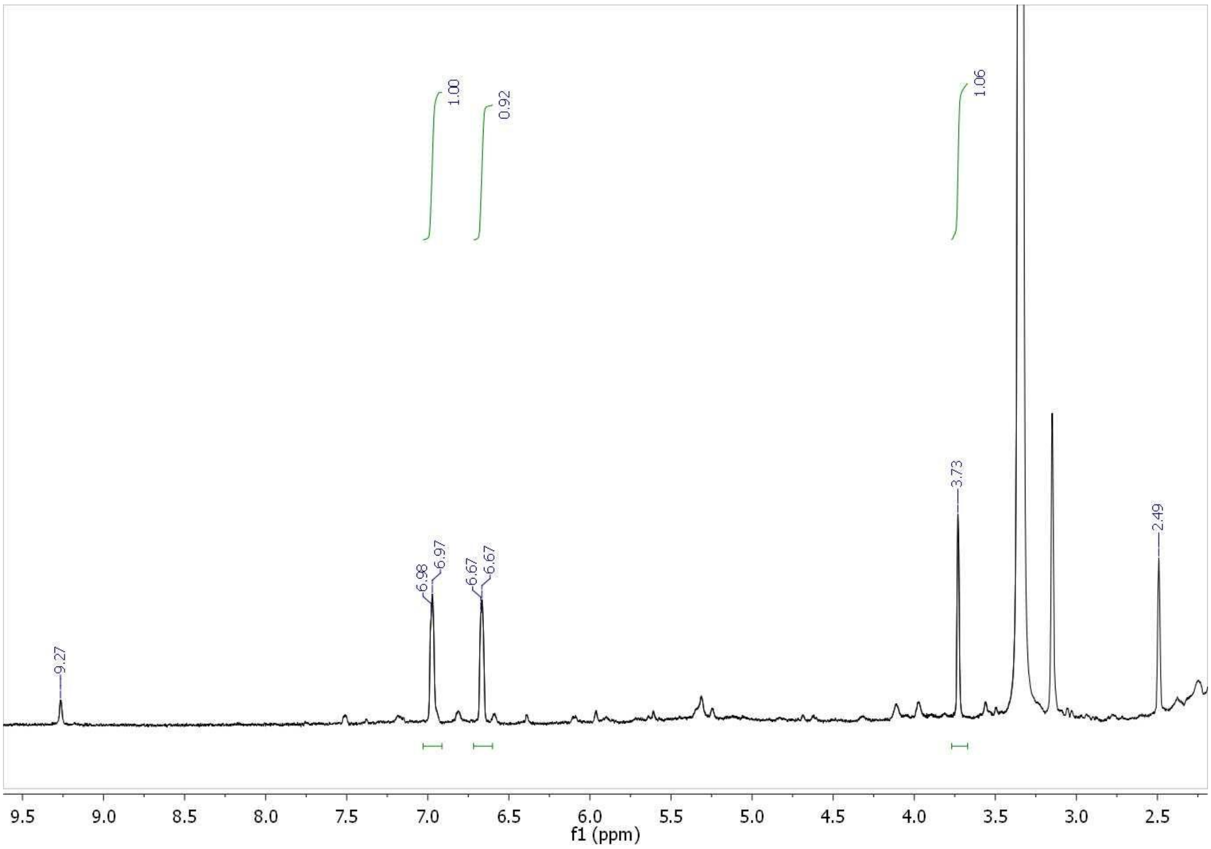

Figure S2. COSY spectrum (600 MHz) of (**1**) in DMSO-d_6.

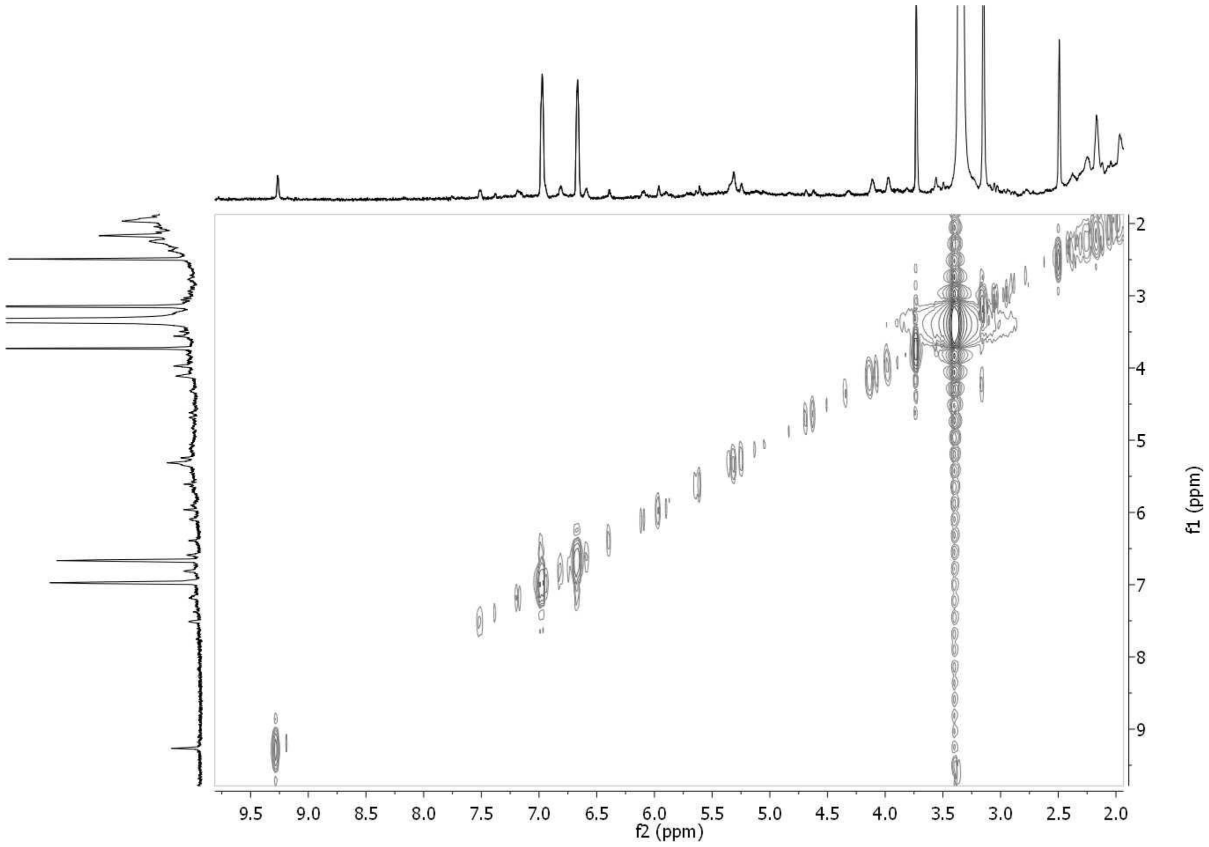

Figure S3. HSQC spectrum (600 MHz) of (**1**) in DMSO-d_6.

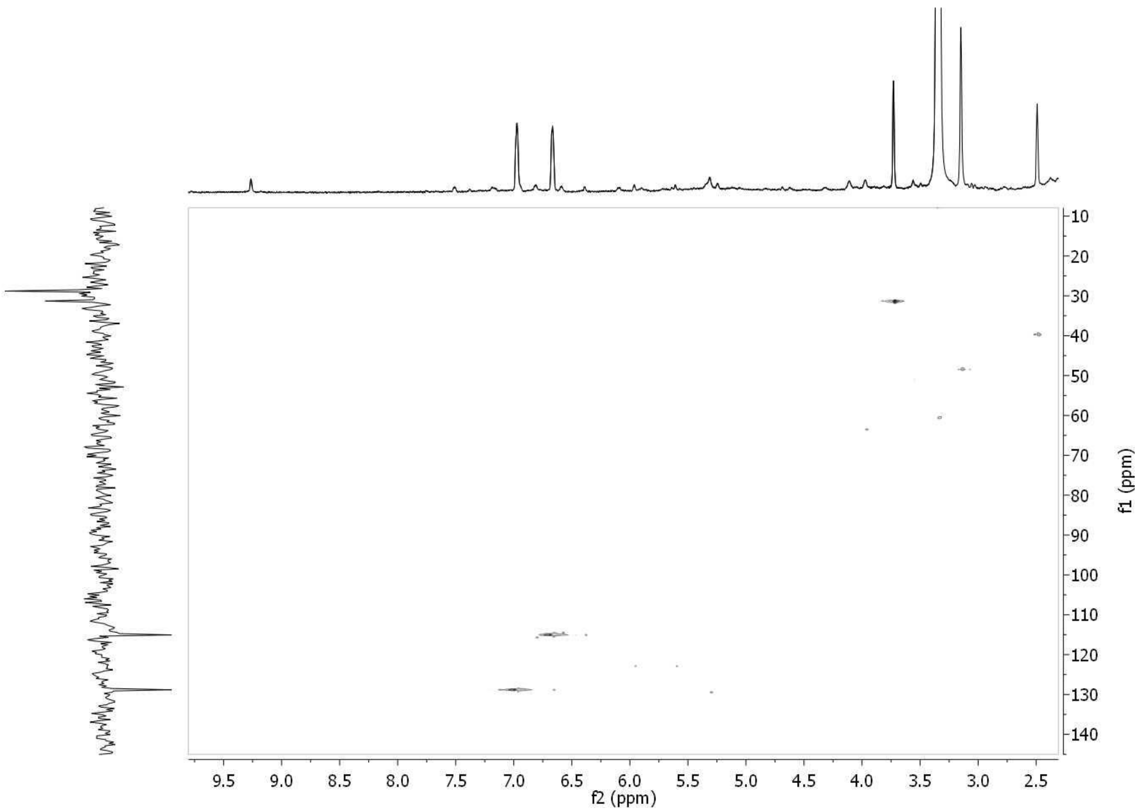

Figure S4. HMBC spectrum (600 MHz) of (**1**) in DMSO-d_6.

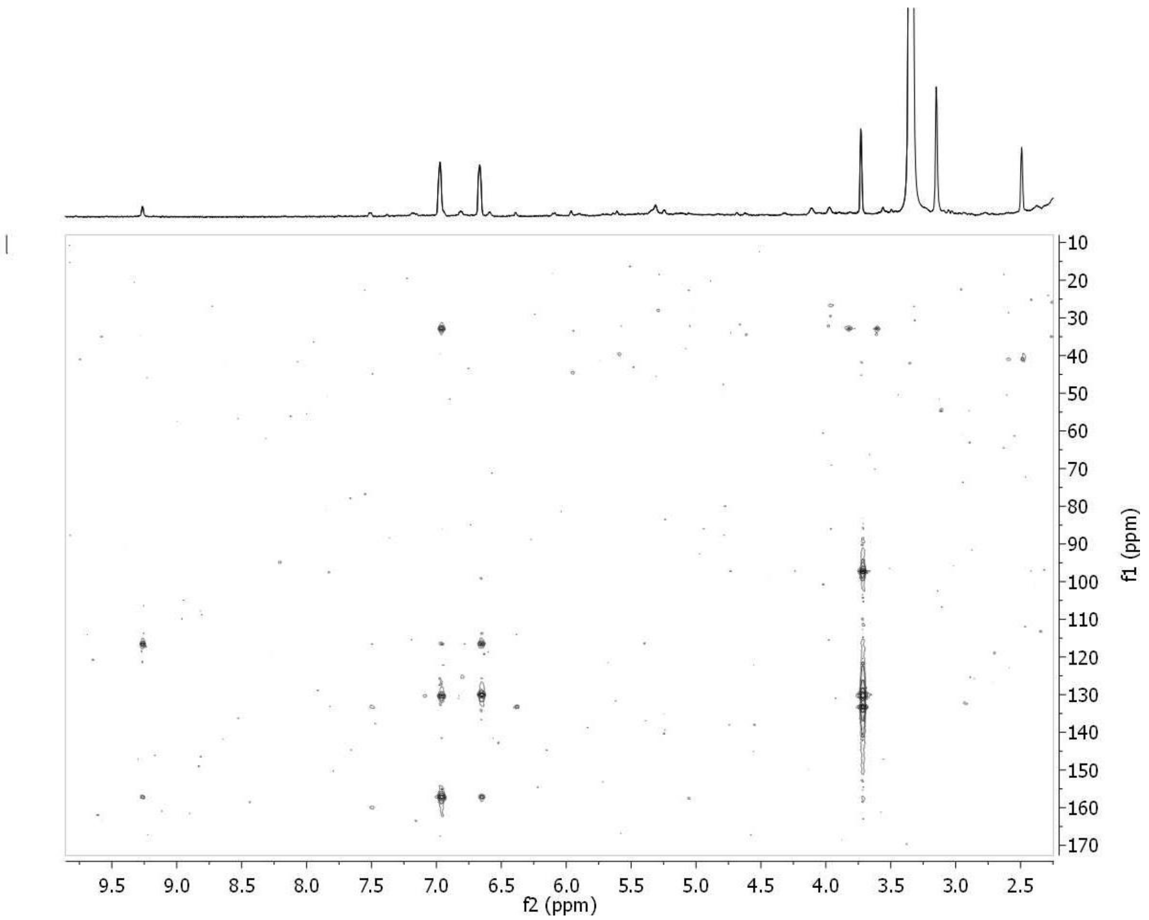

Figure S5. ^{1}H spectrum (600 MHz) of 4′-((3,4,5-tribromo-1*H*-pyrrol-2-yl)methyl)phenol (**1**) in CDCl3.

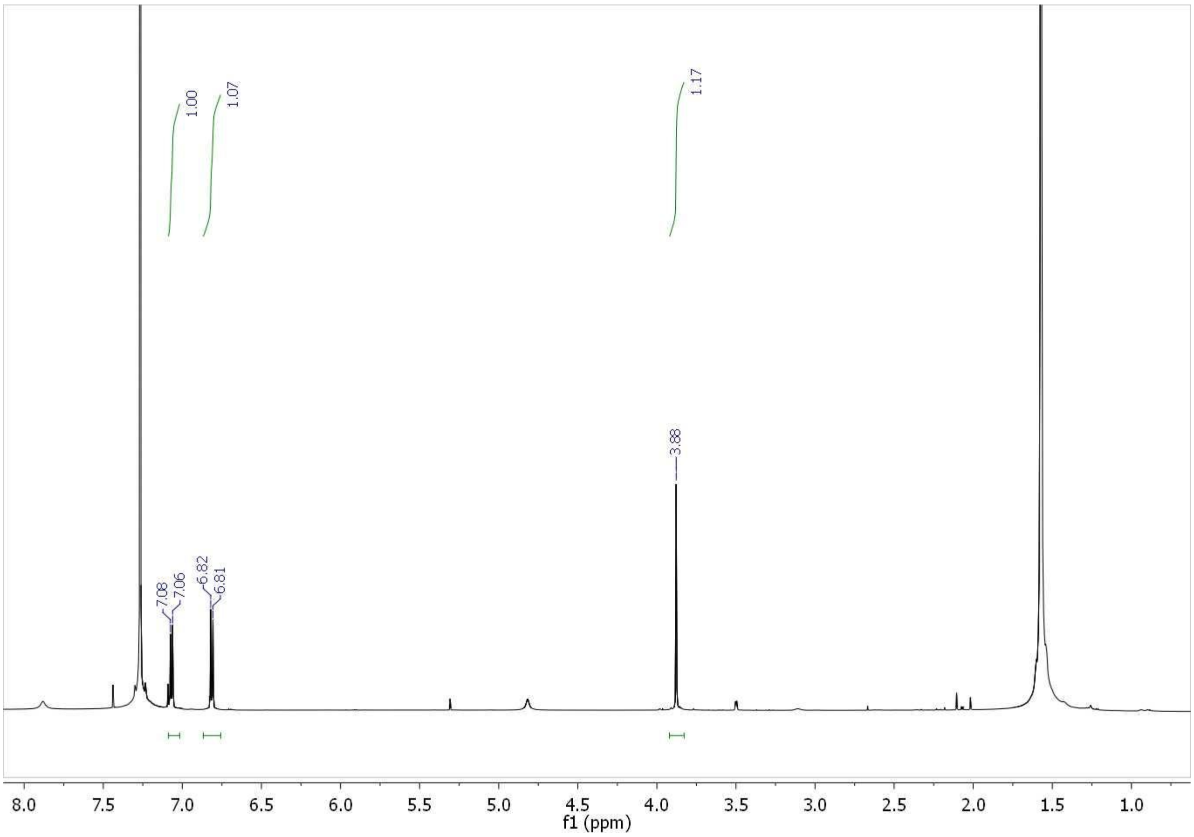

Figure S6. ^{13}C NMR spectrum (125 MHz) of (**1**) in $CDCl_3$.

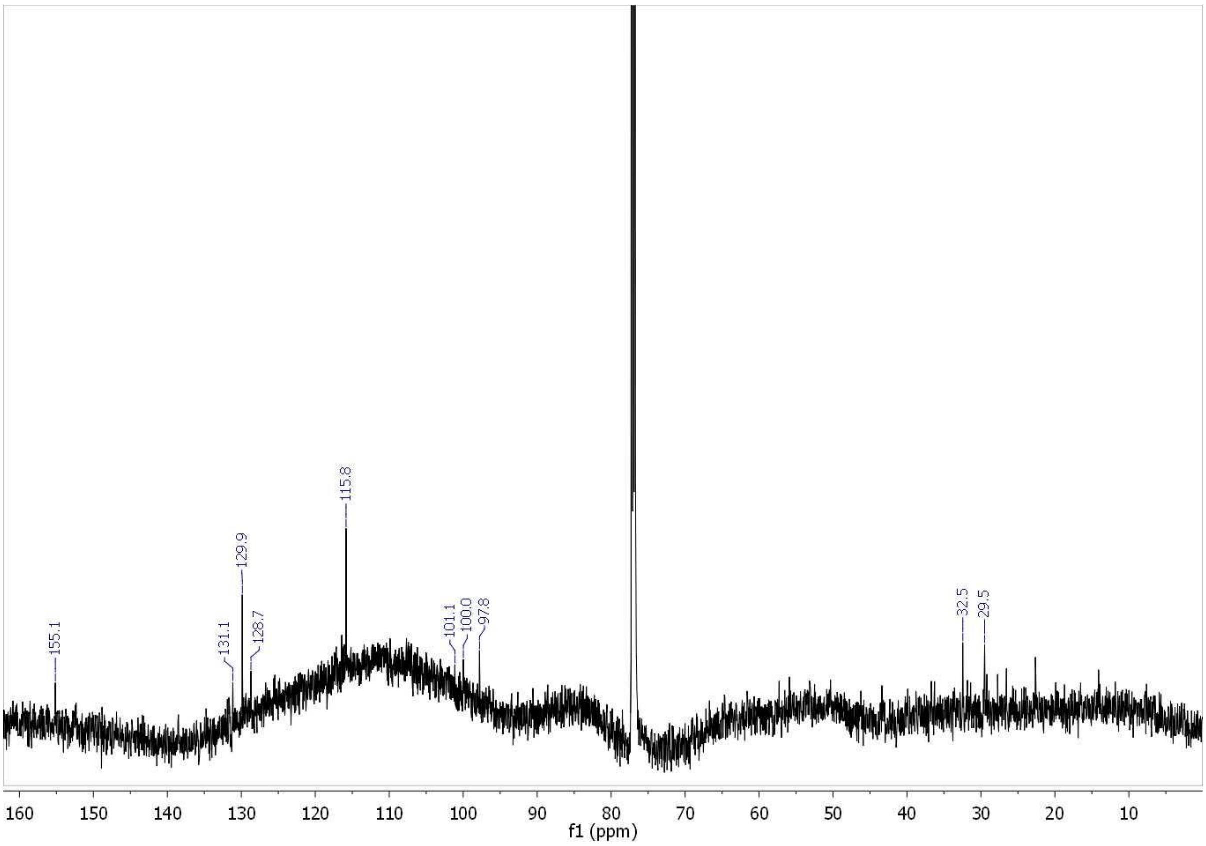

Figure S7. COSY spectrum (600 MHz) of (**1**) in $CDCl_3$.

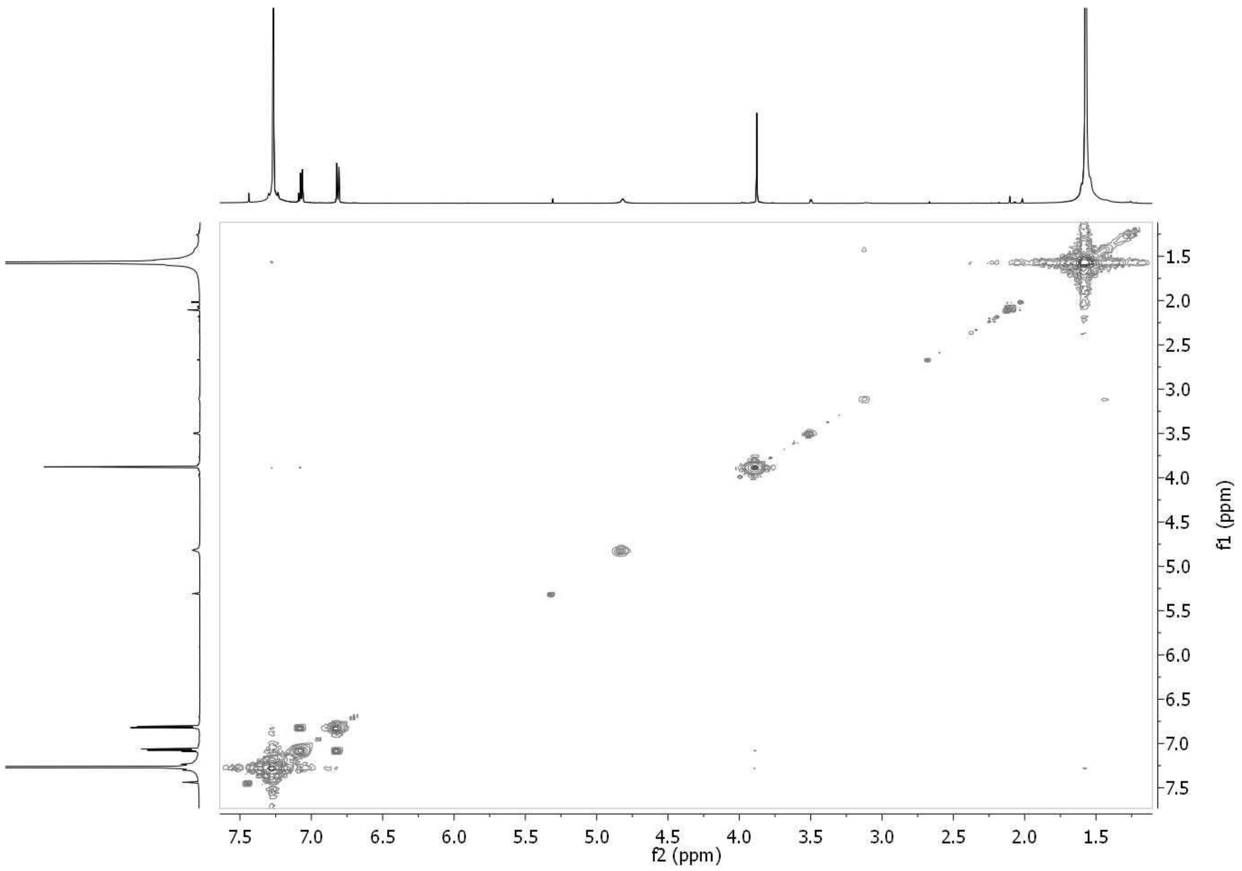

Figure S8. HSQC spectrum (600 MHz) of (**1**) in $CDCl_3$.

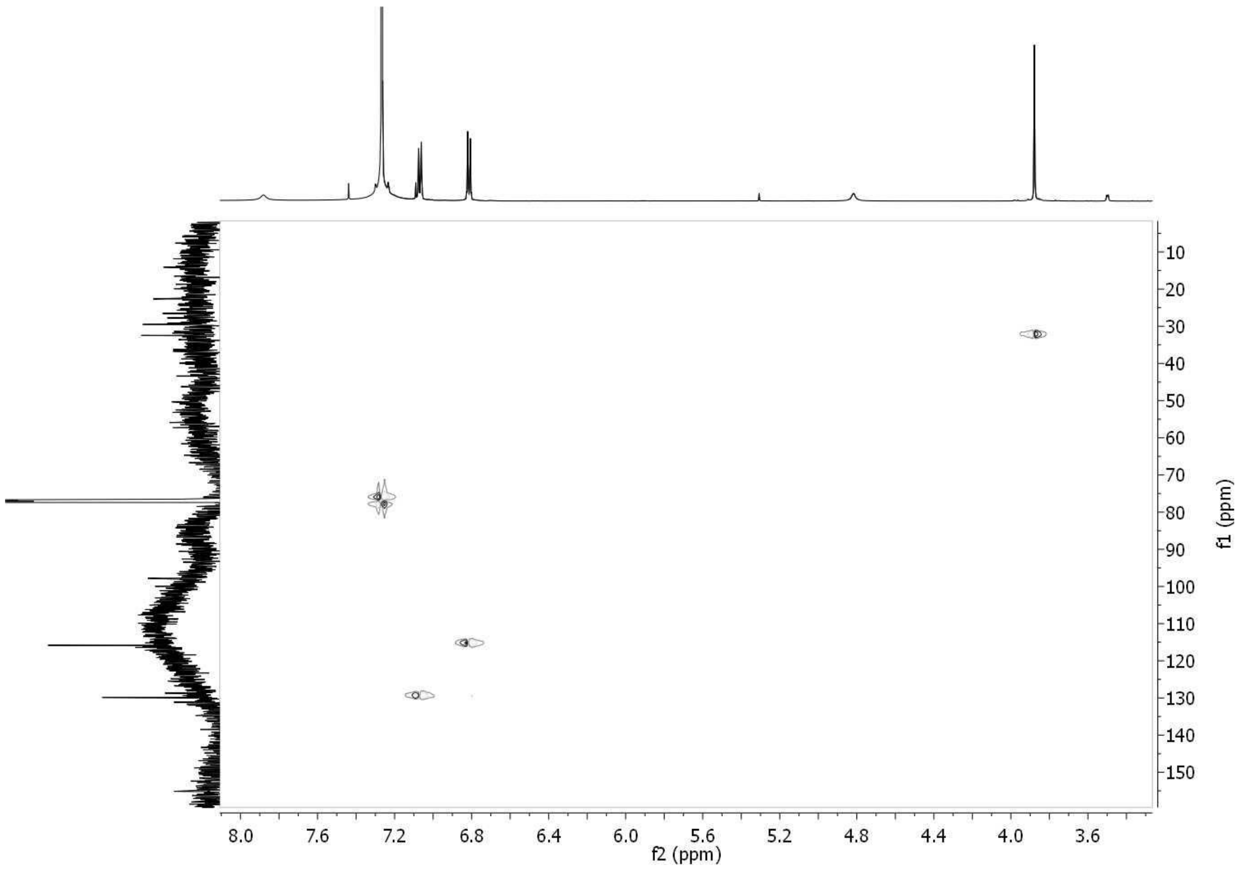

Figure S9. HMBC spectrum (600 MHz) of (**1**) in $CDCl_3$ J_{CH} = 7.5 Hz.

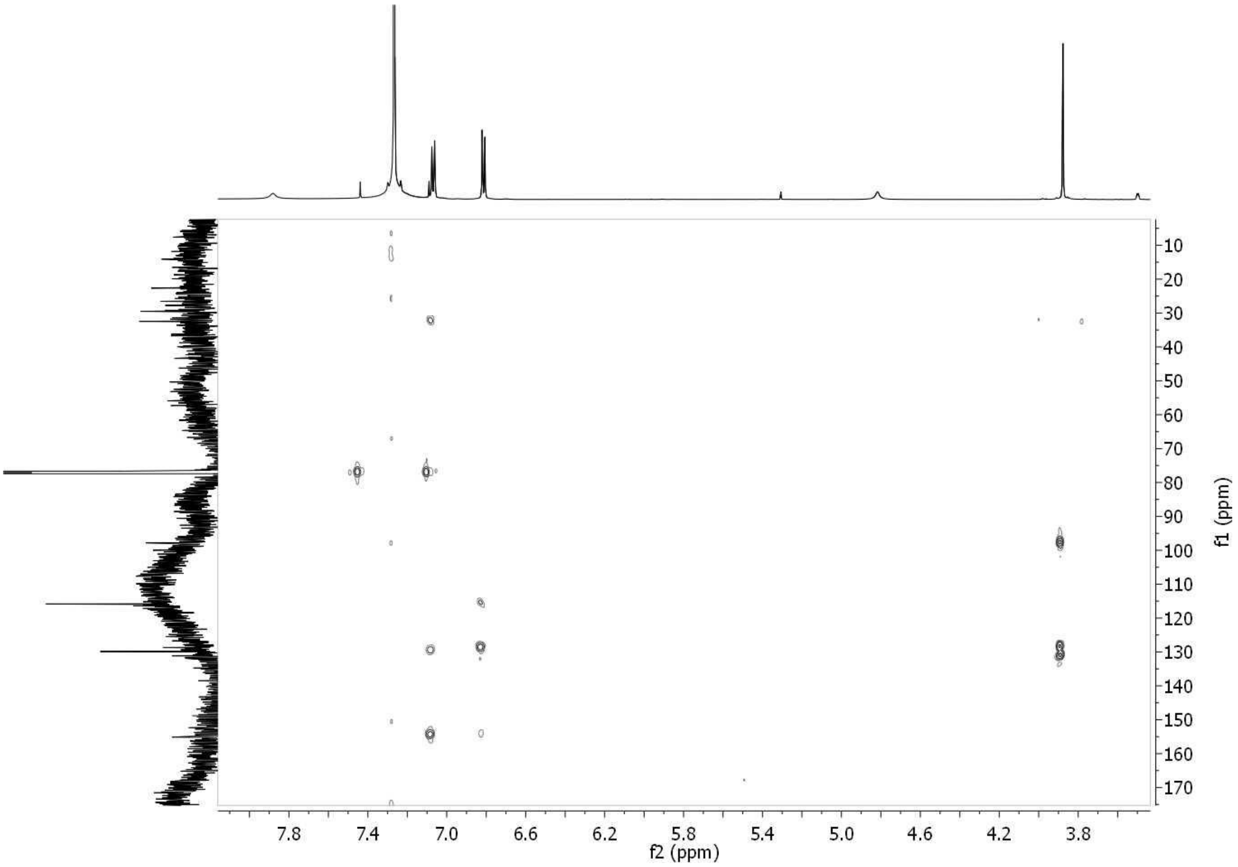

Figure S10. HMBC spectrum (600 MHz) of (**1**) in $CDCl_3$ J_{CH} = 12 Hz.

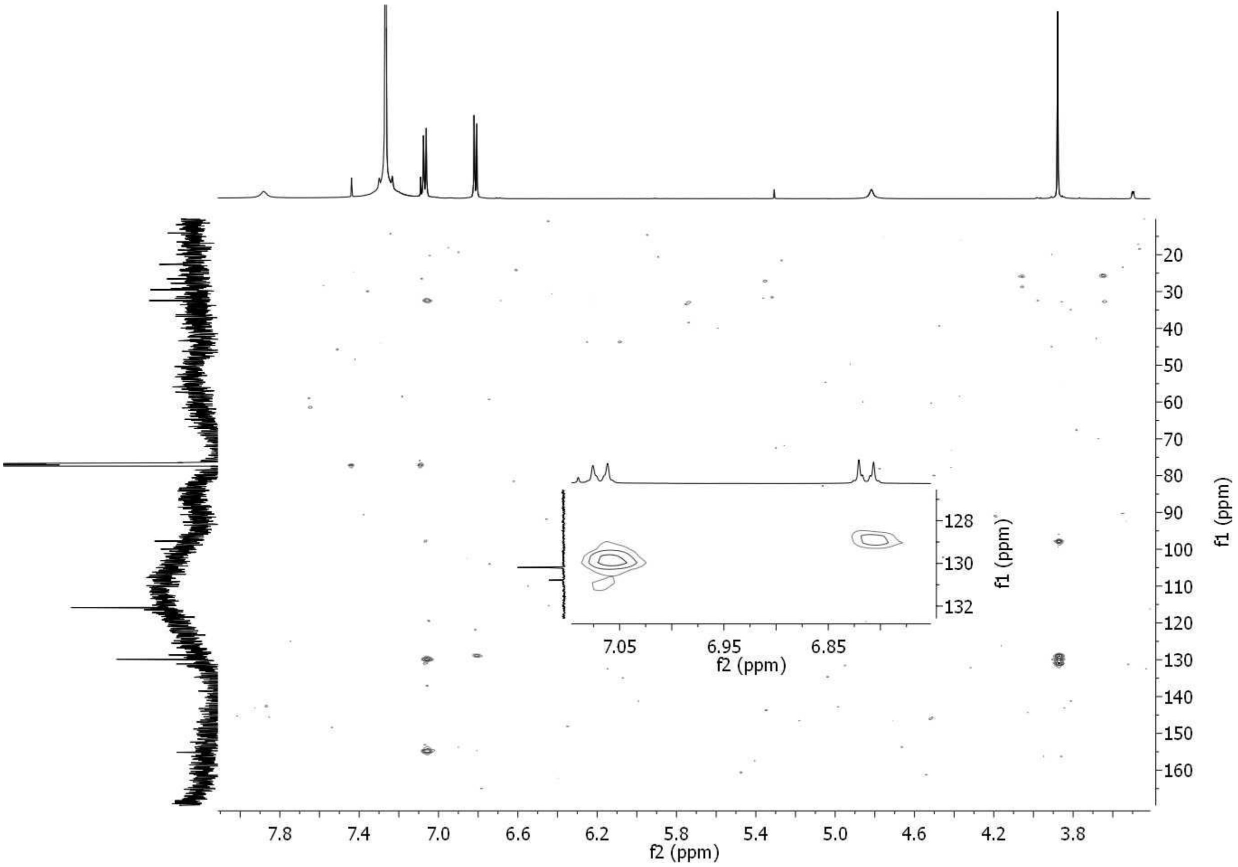

Figure S11. ^{1}H spectrum (600 MHz) of (**1**) in CD_3OD.

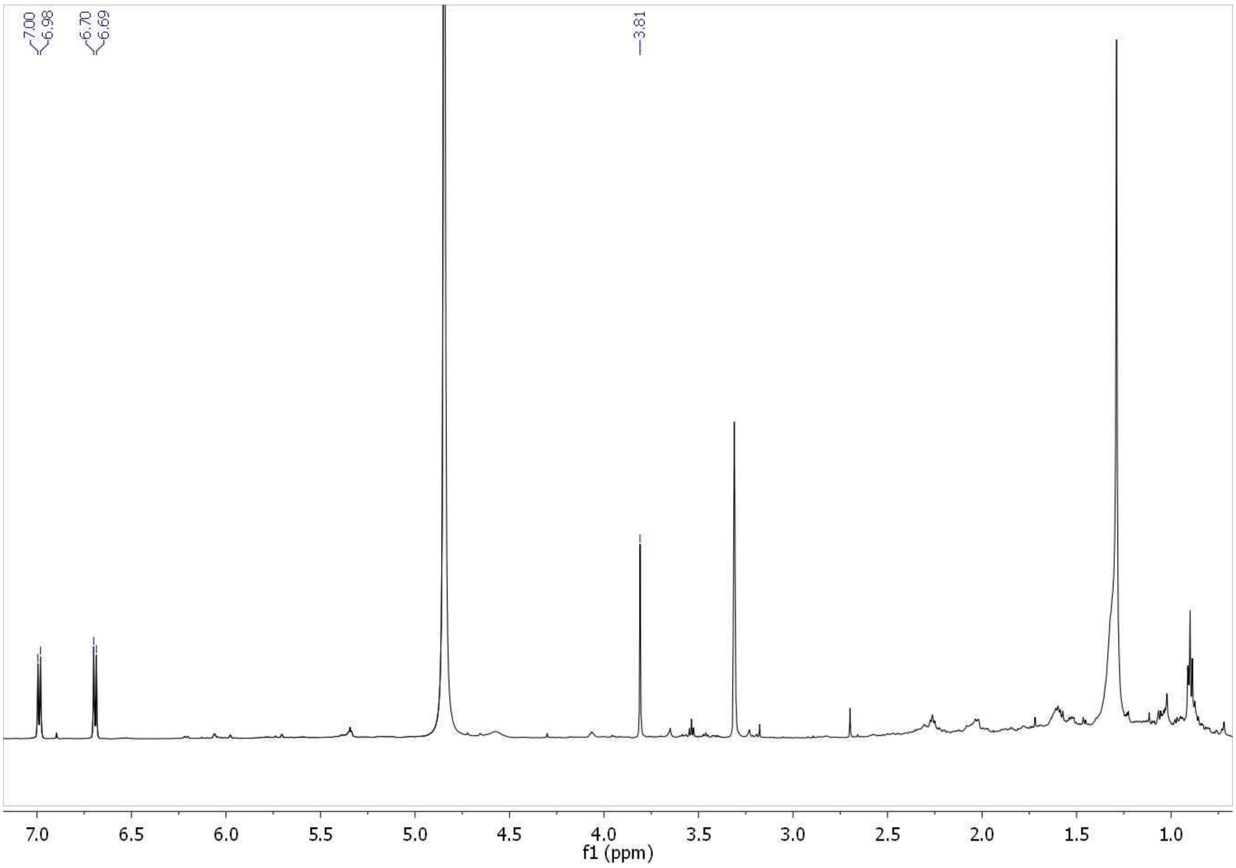

Figure S12. ^{13}C NMR spectrum (125 MHz) of (**1**) in CD_3OD.

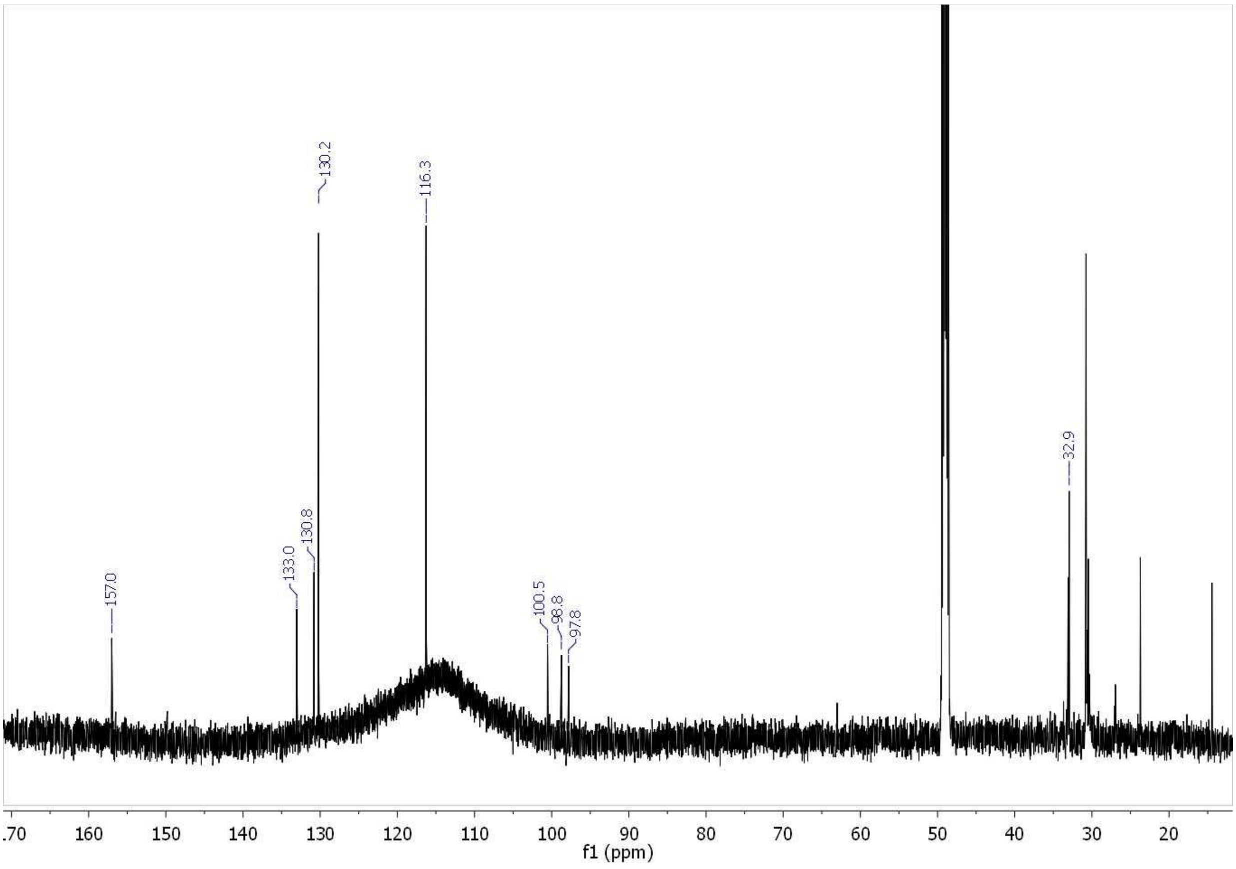

Figure S13. HSQC spectrum (600 MHz) of (**1**) in CD_3OD.

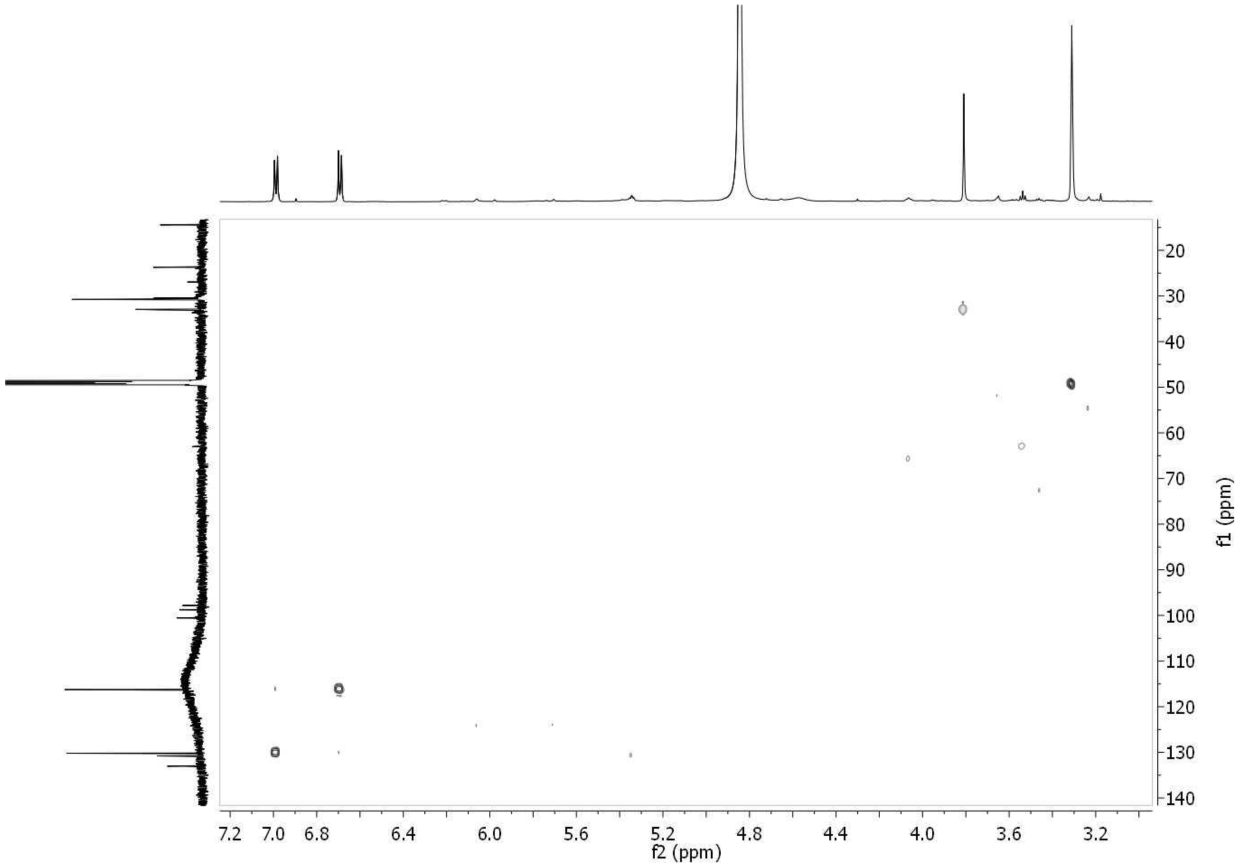

Figure S14. HMBC spectrum (600 MHz) of (**1**) in CD_3OD.

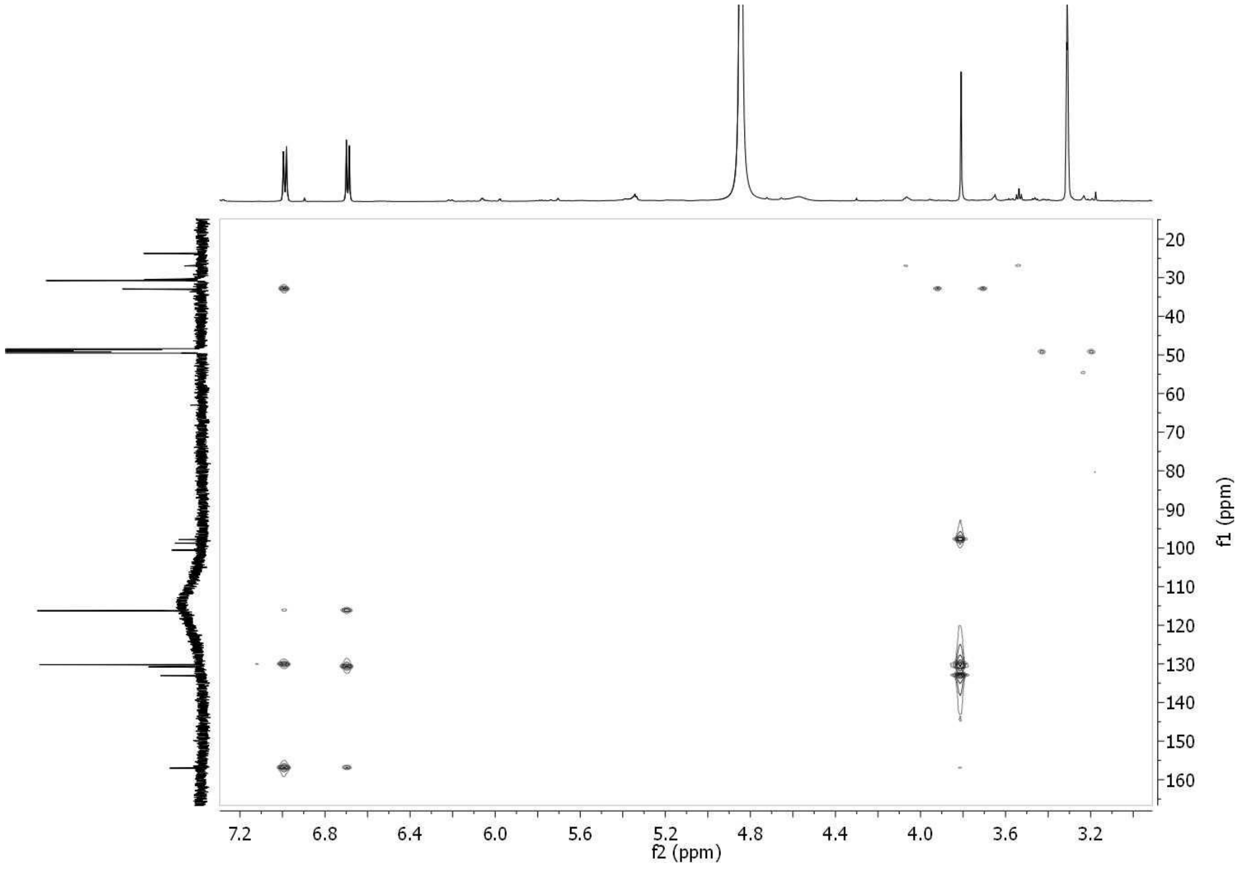

Table S1. NMR data (600 MHz and 125 MHz, Figures S1–S14) for 4′-((3,4,5-tribromo-1*H*-pyrrol-2-yl)methyl)phenol (**1**).

**Position**	**DMSO-*d*$_6$**				**$CDCl_3$**				**CD_3OD**			
	**δ_C, mult.** [a]	**δ_H (*J* Hz)**	**gCOSY**	**gHMBC**	**δ_C, mult.**	**δ_H (*J* Hz)**	**gCOSY**	**gHMBC**	**δ_C, mult.**	**δ_H (*J* Hz)**	**gCOSY**	**gHMBC**
NH	-	-	-	-	-	-	-	-	-	-	-	-
2	132.3, qC	-	-	-	131.1, qC	-	-	-	133.0, qC	-	-	-
3	96.3, qC	-	-	-	97.8, qC	-	-	-	97.8, qC	-	-	-
4	nd [b]	-	-	-	100.0, qC	-	-	-	100.5, qC	-	-	-
5	nd [b]	-	-	-	100.0, qC	-	-		98.8, qC	-	-	-
6	31.8, CH_2	3.73, s	-	2, 7, 8, 12	32.5, CH_2	3.87, s	-	2, 7, 8, 12	32.9, CH_2	3.87, s	-	2, 7, 8, 12
7	129.2, qC	-	-		128.7, qC	-			130.8, qC	-	-	-
8	128.8, CH	6.97, d (8.2)	9	6, 7, 9, 10, 11	129.9, CH	7.05, d (8.3)	9	6, 7, 9, 10, 11	130.2, CH	7.05, d (8.3)	9	6, 7, 9, 10, 11
9	115.3, CH	6.67, d (8.2)	8	8, 10, 11, 12	115.8, CH	6.81, d (8.3)	8	8, 10, 11, 12	116.3, CH	6.81, d (8.3)	8	8, 10, 11, 12
10	155.9, qC	-	-	-	155.1, qC	-	-	-	157.0, qC	-	-	-
OH	-	-	-	-	-	-	-	-	-	-	-	-
11	115.3, CH	6.67, d (8.2)	12	8, 10, 11, 12	115.8, CH	6.81, d (8.3)	12	8, 10, 11, 12	116.3, CH	6.81, d (8.3)	12	8, 10, 11, 12
12	128.8, CH	6.97, d (8.2)	11	6, 8, 9, 10, 11	129.9, CH	7.05, d (8.3)	11	6, 8, 9, 10, 11	130.2, CH	7.05, d (8.3)	11	6, 8, 9, 10, 11

[a] carbon shifts from HSQC and HMBC; [b] nd = not detected.

Figure S15. ^{1}H spectrum (600 MHz) of Korormicin G (**2**) in $CDCl_3$.

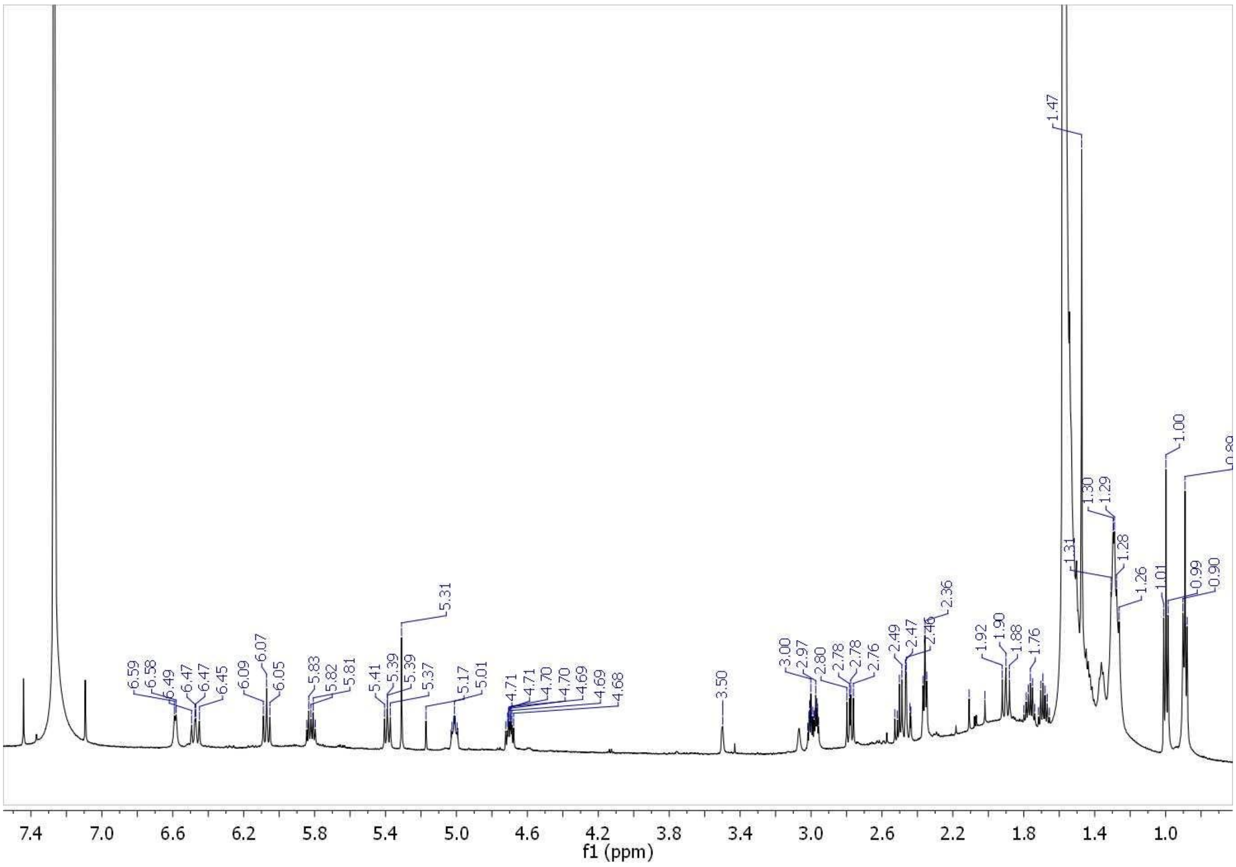

Figure S16. COSY spectrum (600 MHz) of (**2**) in $CDCl_3$.

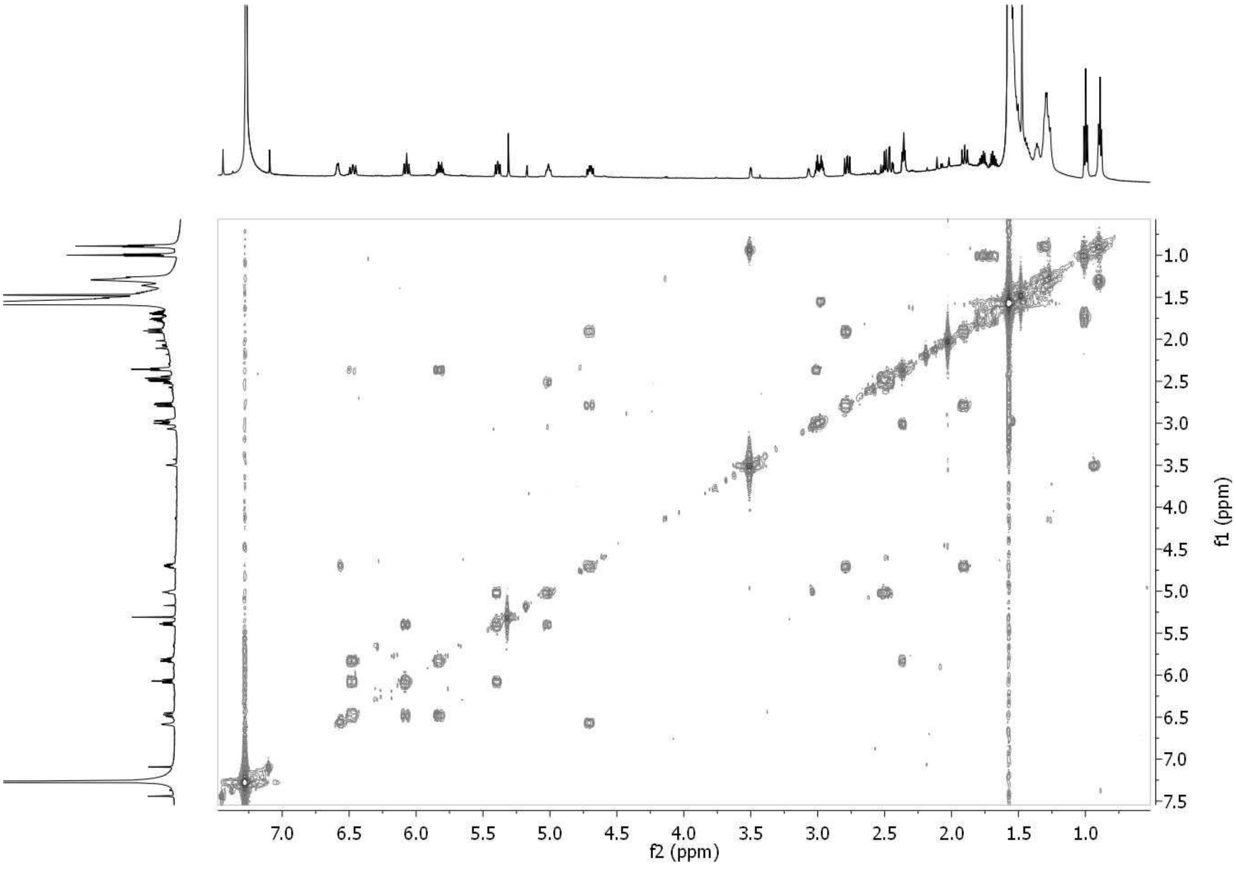

Figure S17. HSQC spectrum (600 MHz) of (**2**) in $CDCl_3$.

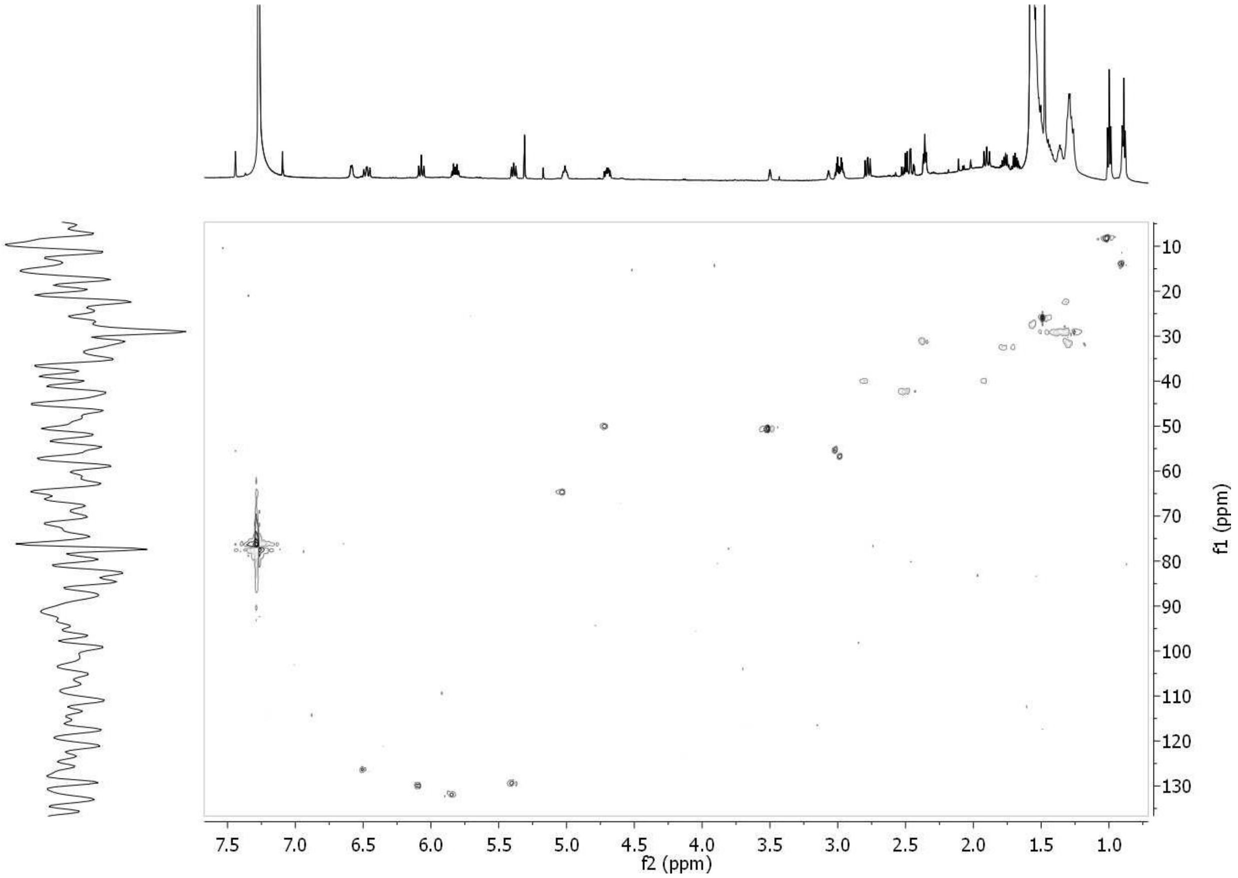

Figure S18. HMBC spectrum (600 MHz) of (**2**) in $CDCl_3$.

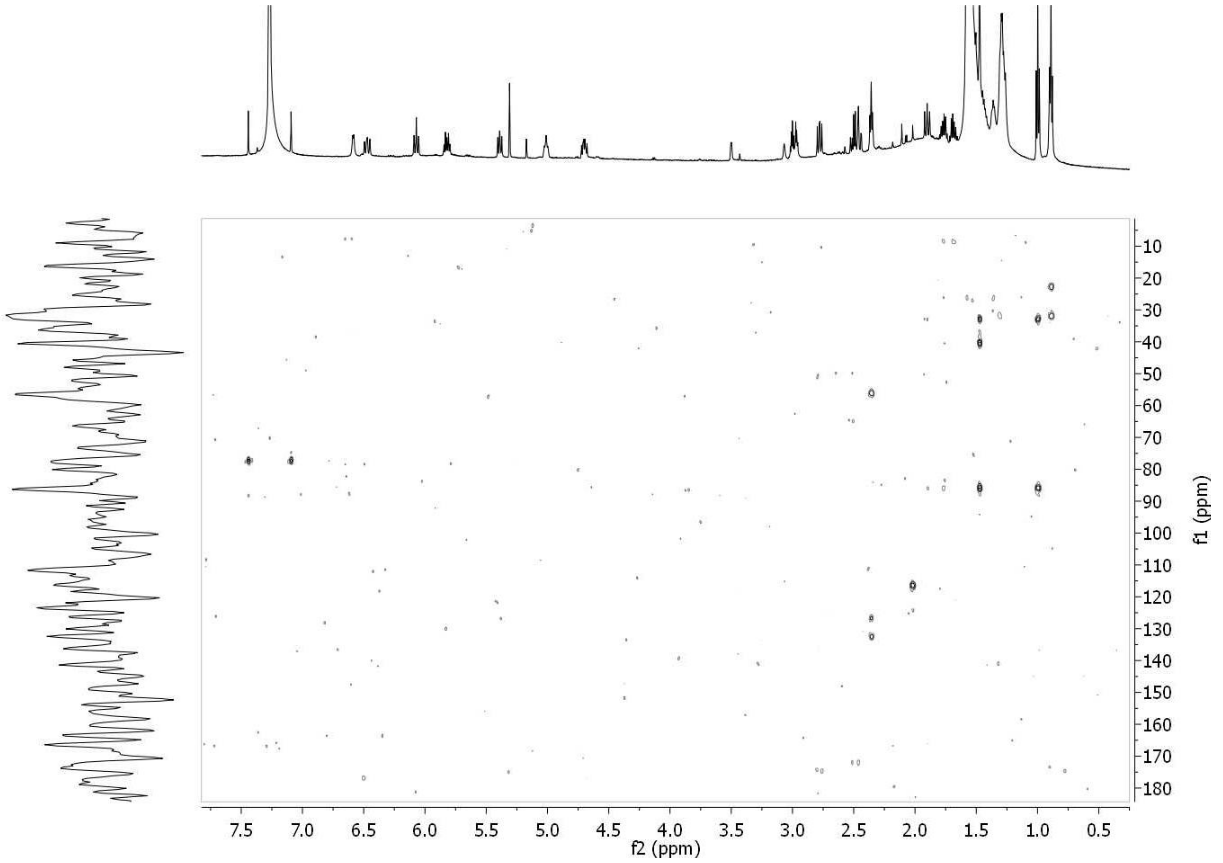

Figure S19. ^{1}H spectrum (600 MHz) of Korormicin H (**3**) in DMSO-d_6.

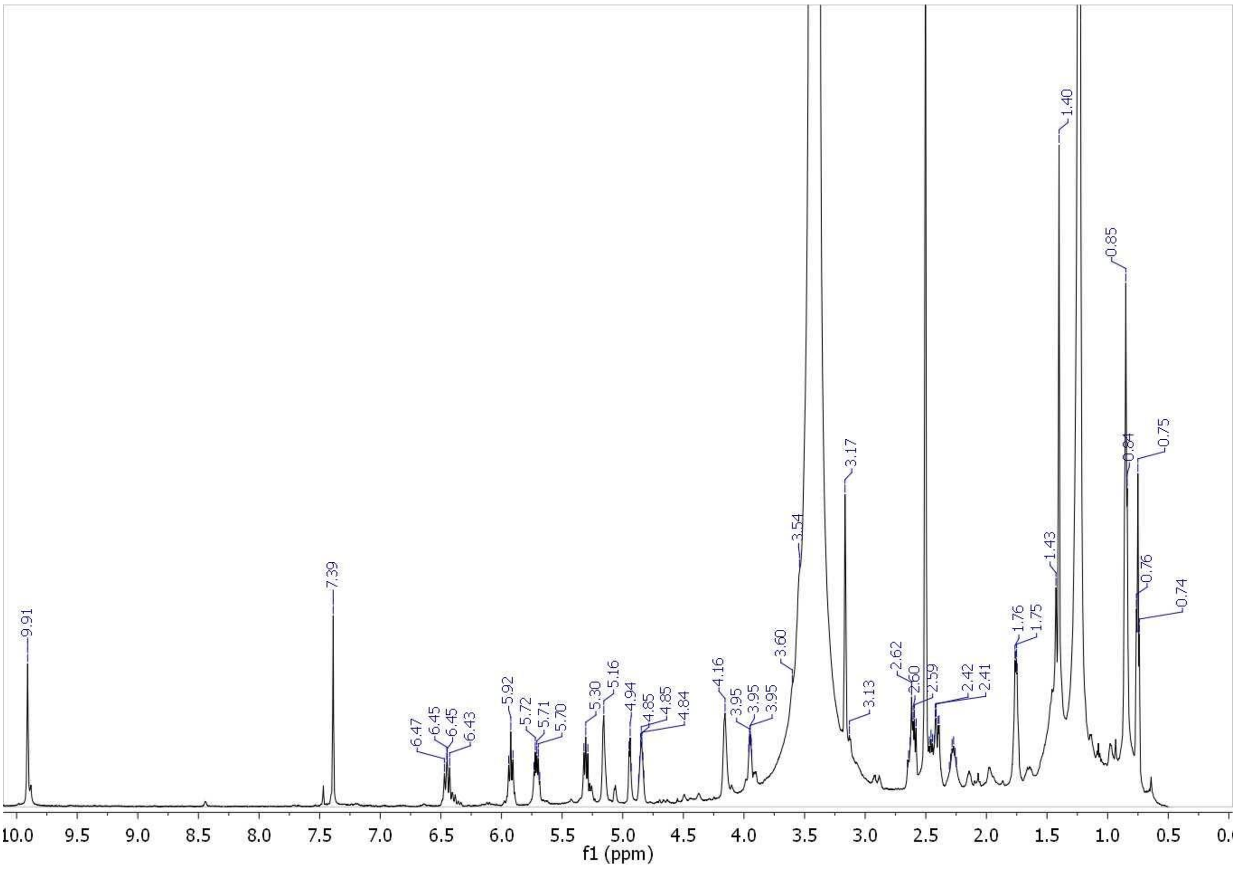

Figure S20. ^{13}C NMR spectrum (125 MHz) of (**2**) in DMSO-d_6.

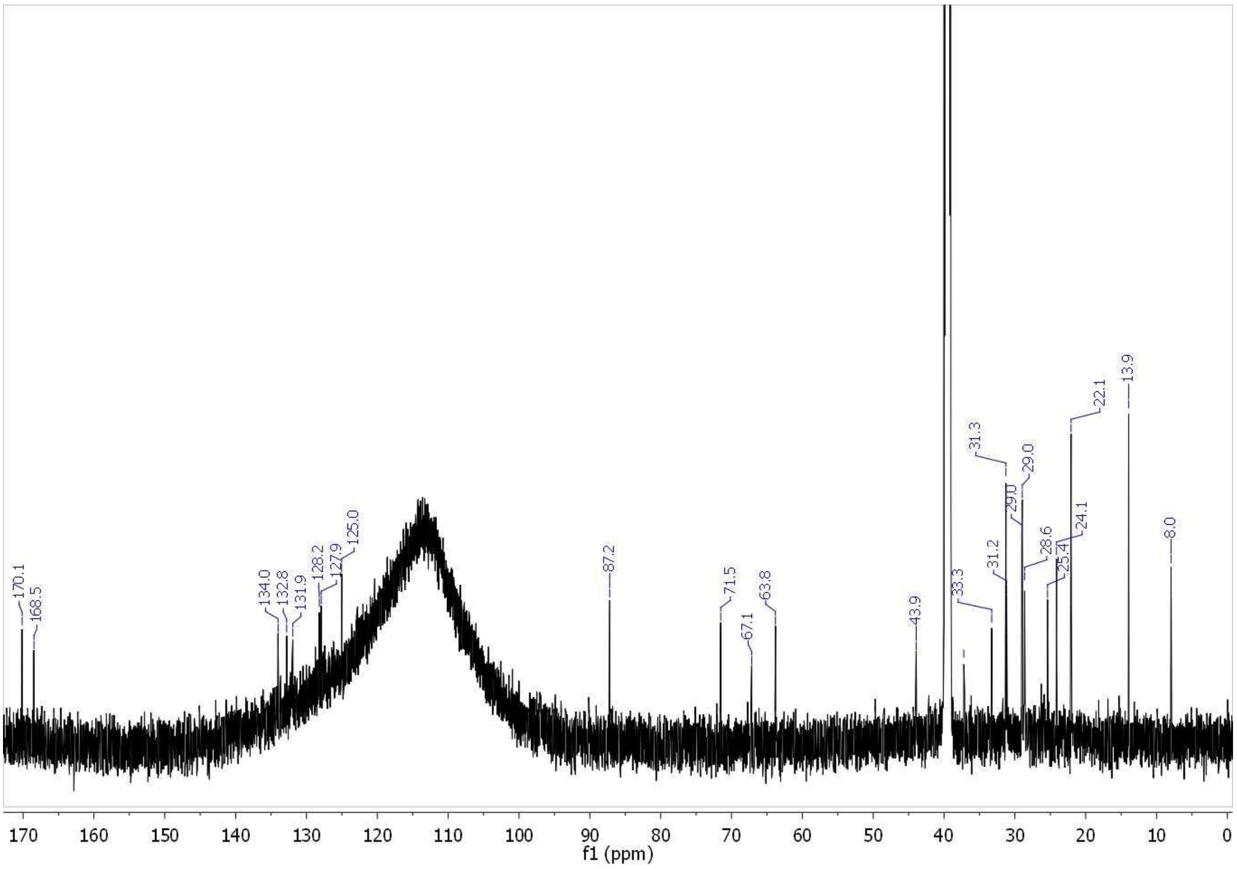

Figure S21. COSY spectrum (600 MHz) of (**3**) in DMSO-d_6.

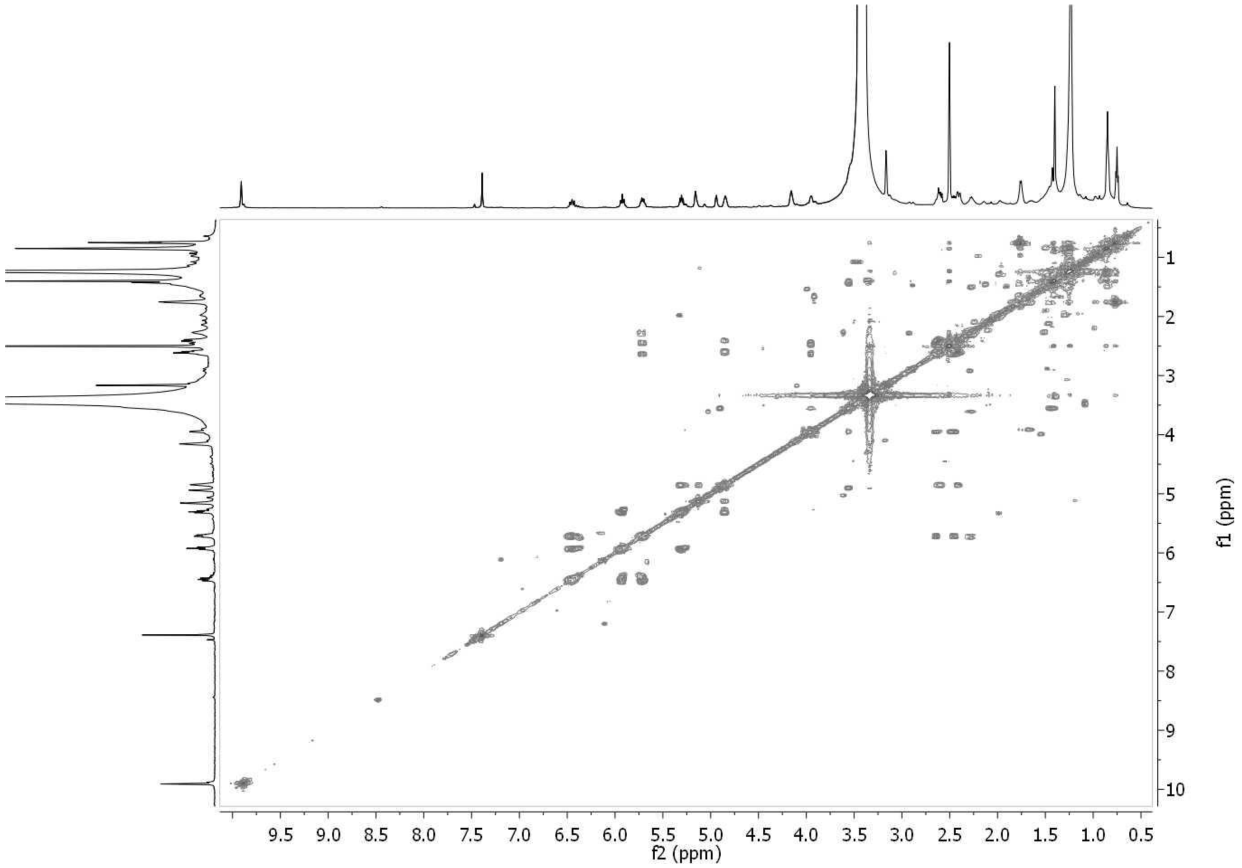

Figure S22. HSQC spectrum (600 MHz) of (**3**) in DMSO-d_6.

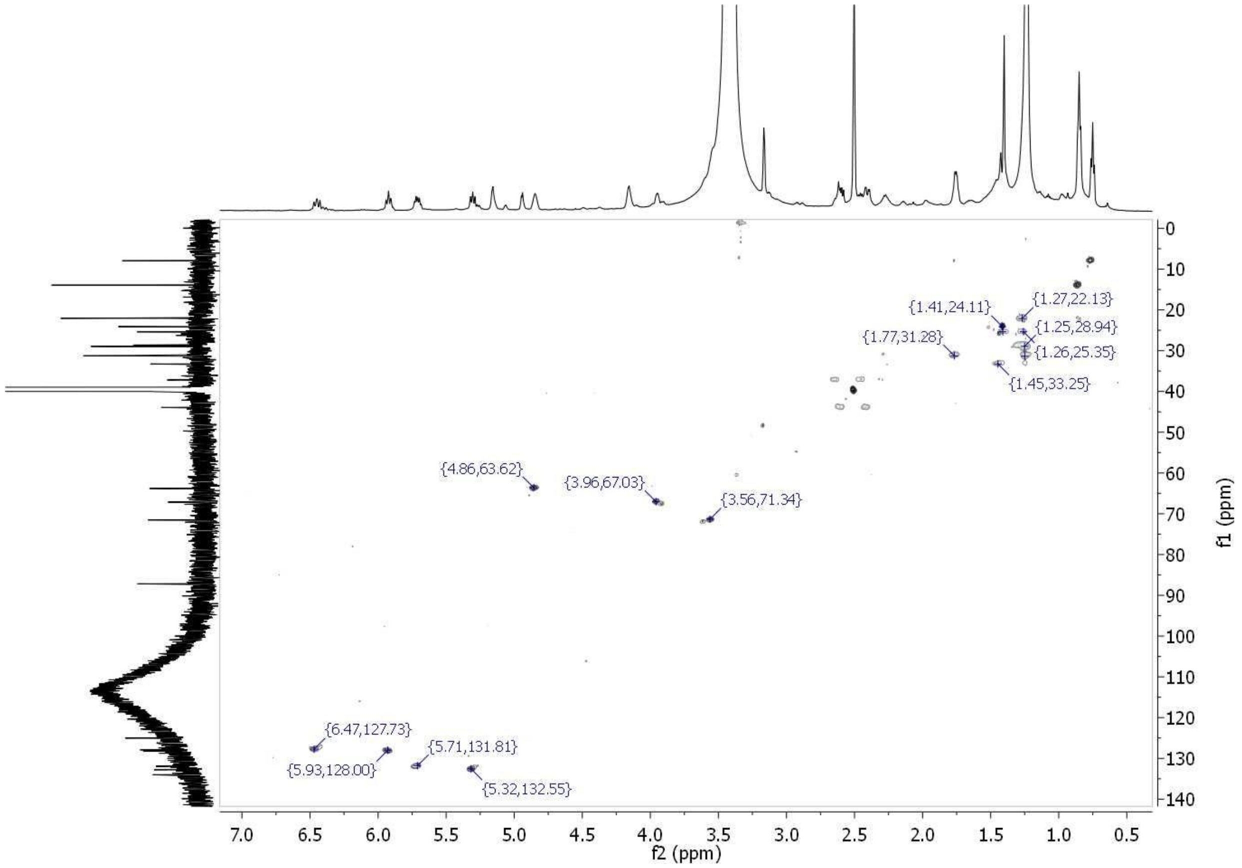

Figure S23. HMBC spectrum (600 MHz) of (**3**) in DMSO-d_6.

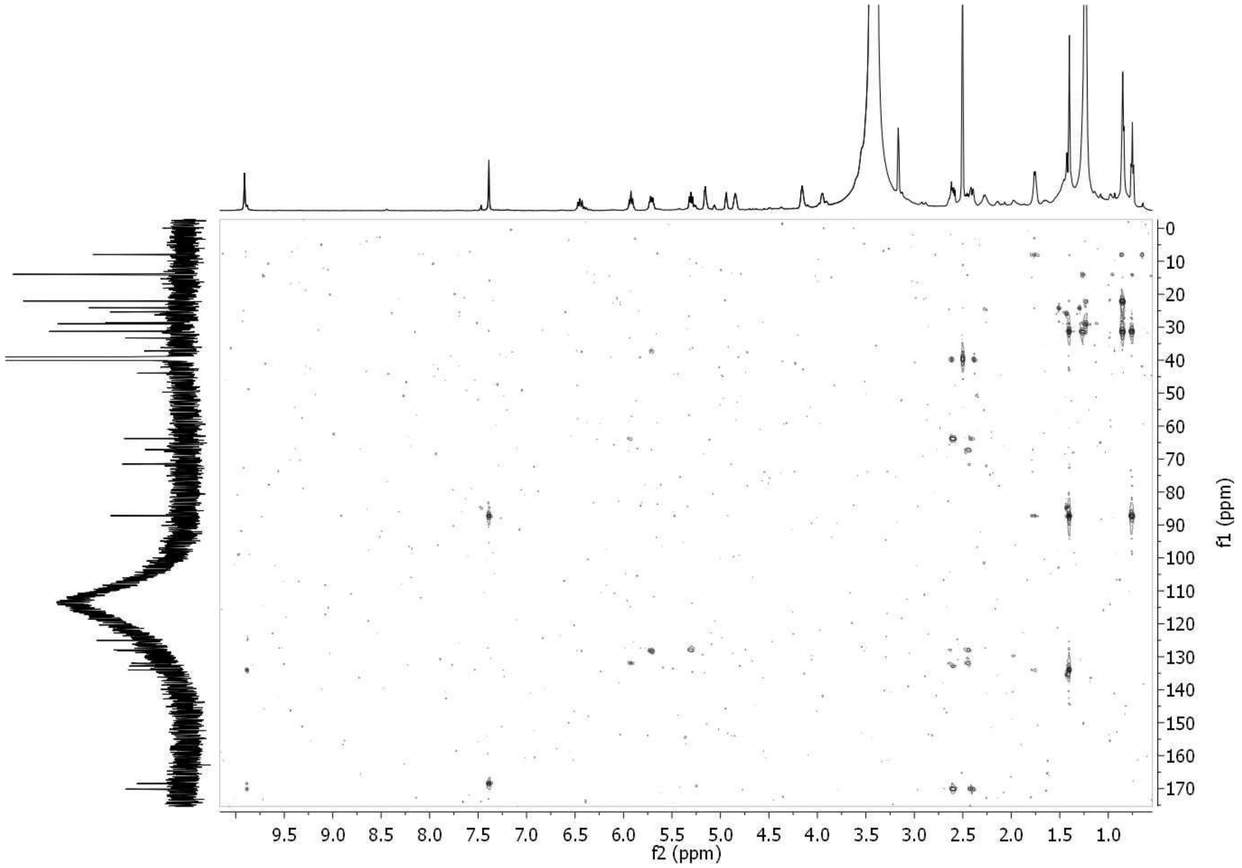

Figure S24. ^{1}H spectrum (600 MHz) of Korormicin I (**4**) in $CDCl_3$.

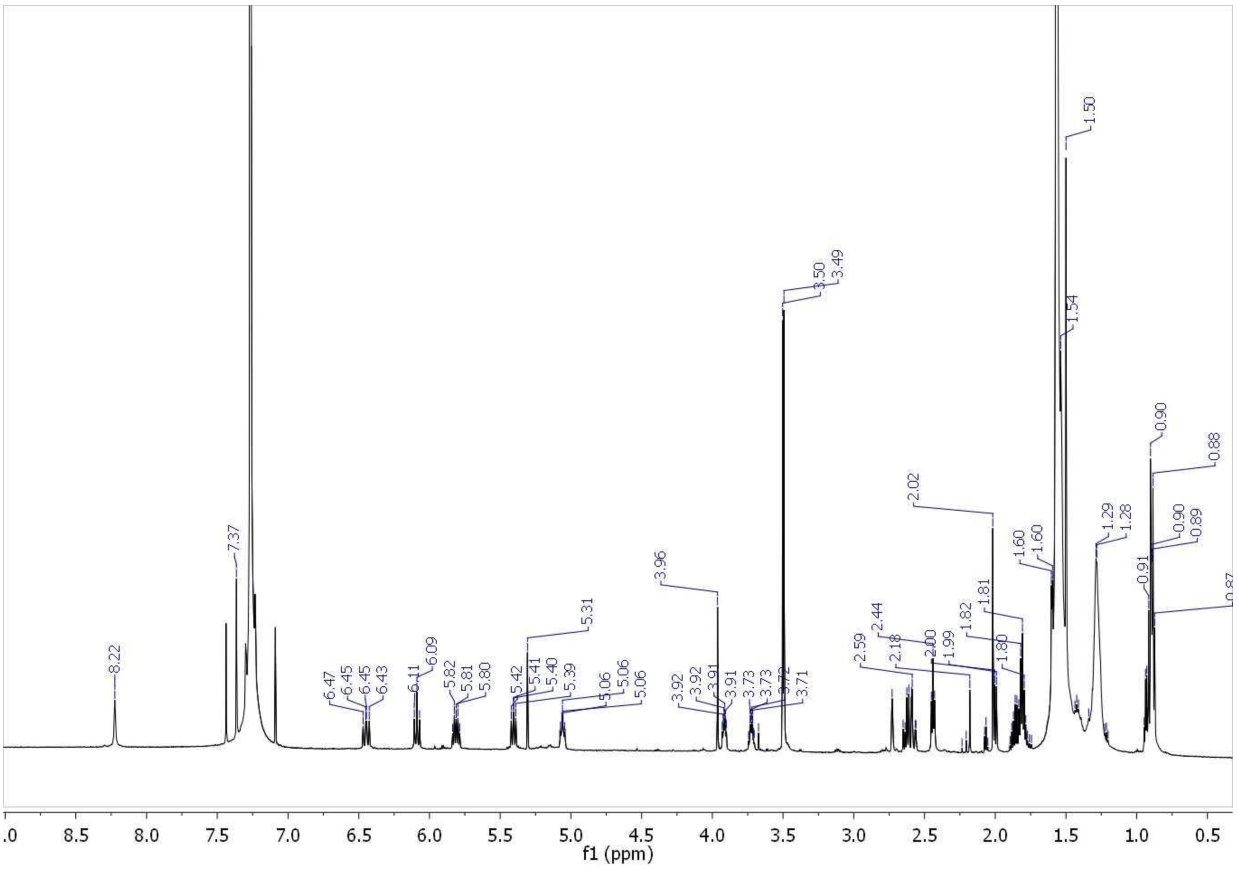

Figure S25. COSY spectrum (600 MHz) of (**4**) in $CDCl_3$.

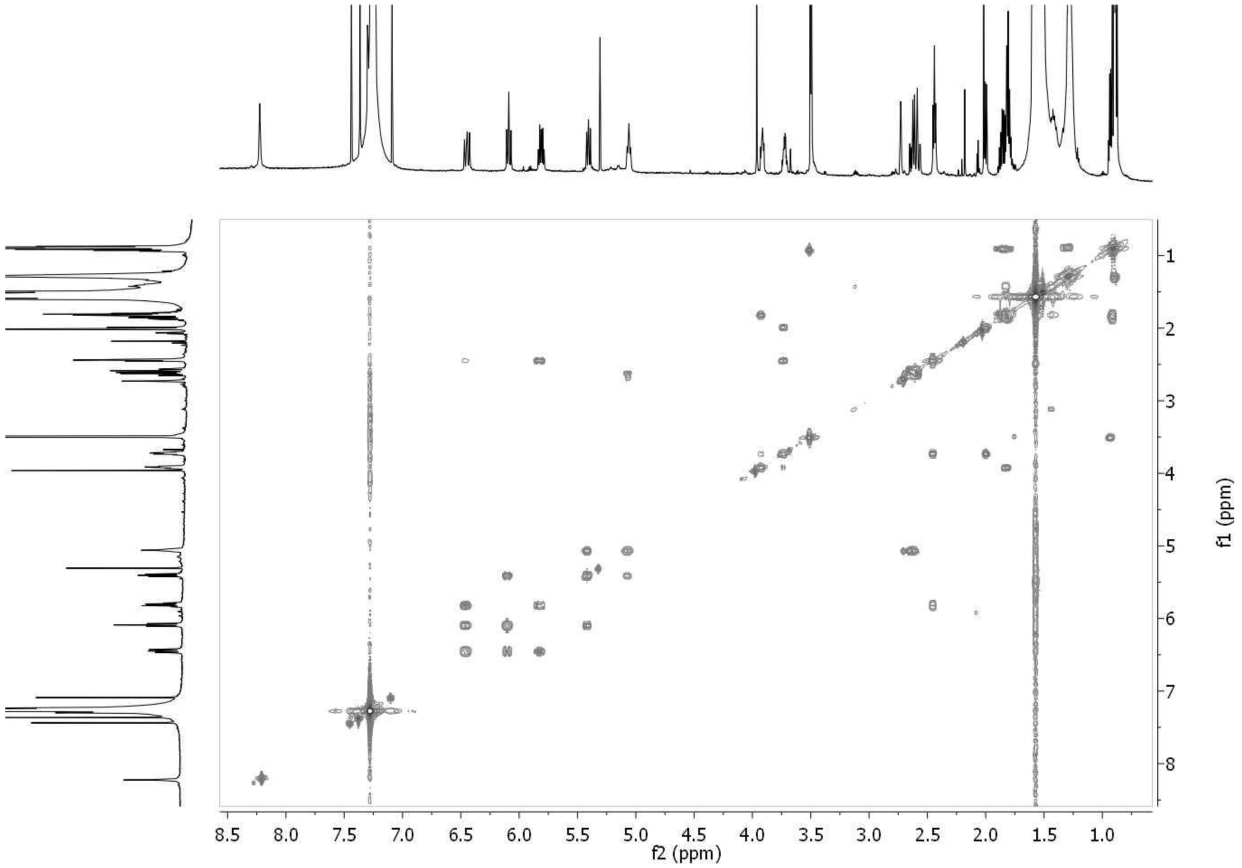

Figure S26. HSQC spectrum (600 MHz) of (**4**) in $CDCl_3$.

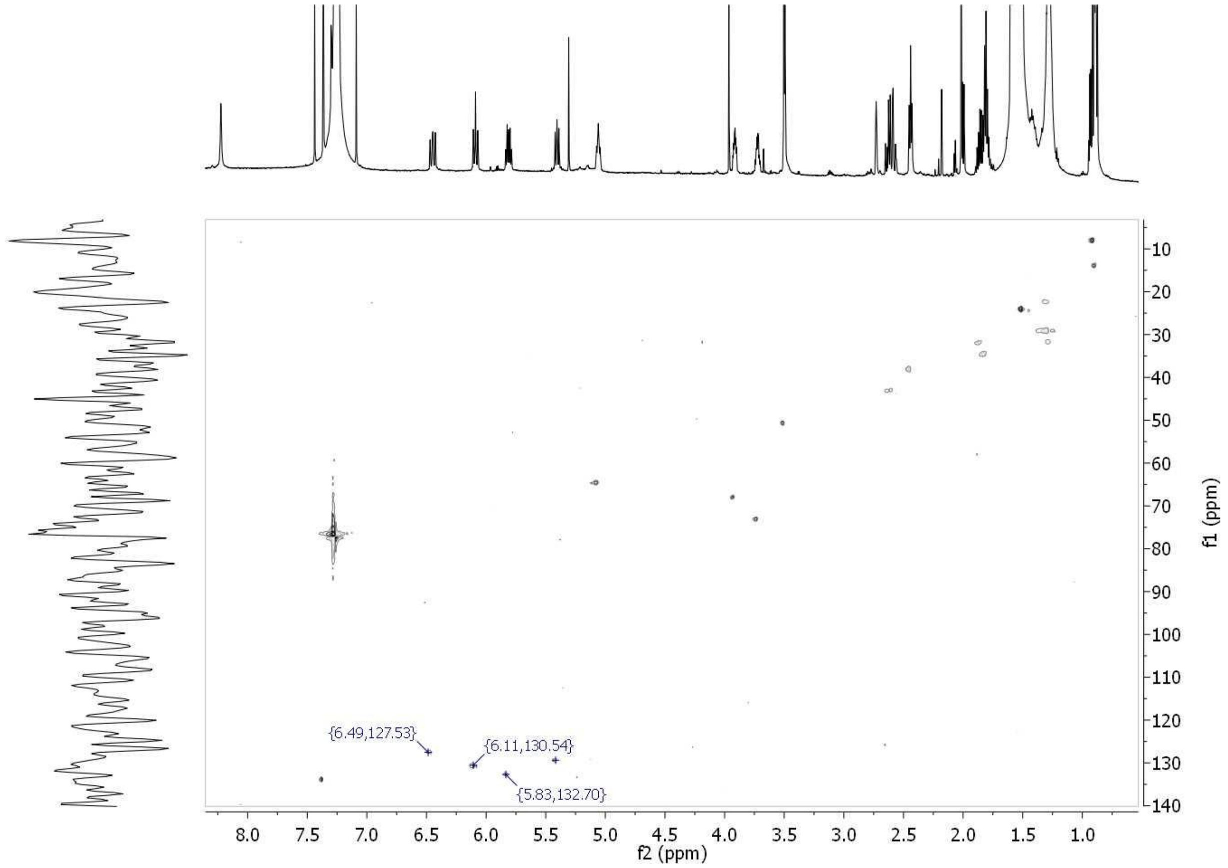

Figure S27. ^{1}H spectrum (600 MHz) of Korormicin I (**4**) in DMSO-d_6.

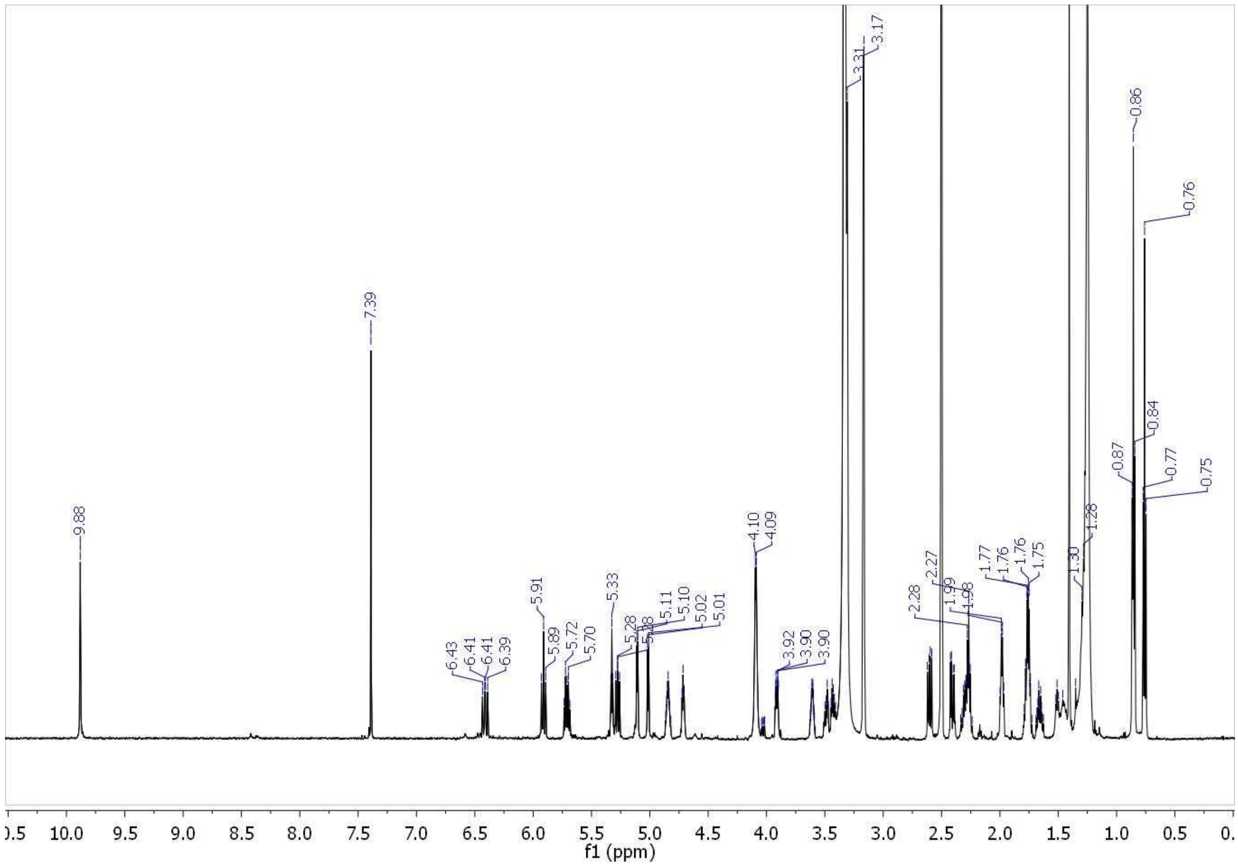

Figure S28. ^{13}C NMR spectrum (125 MHz) of (**4**) in DMSO-d_6.

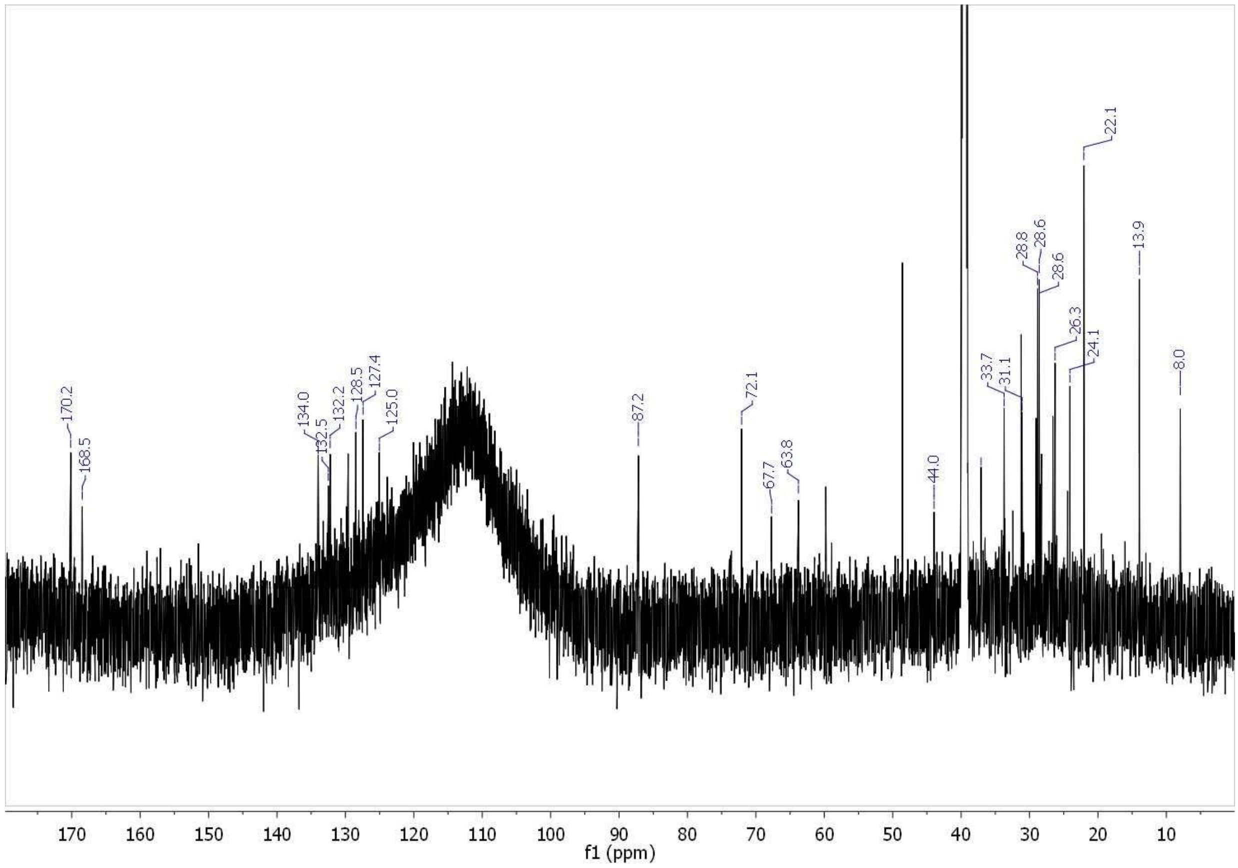

Figure S29. COSY spectrum (600 MHz) of (**4**) in DMSO-d_6.

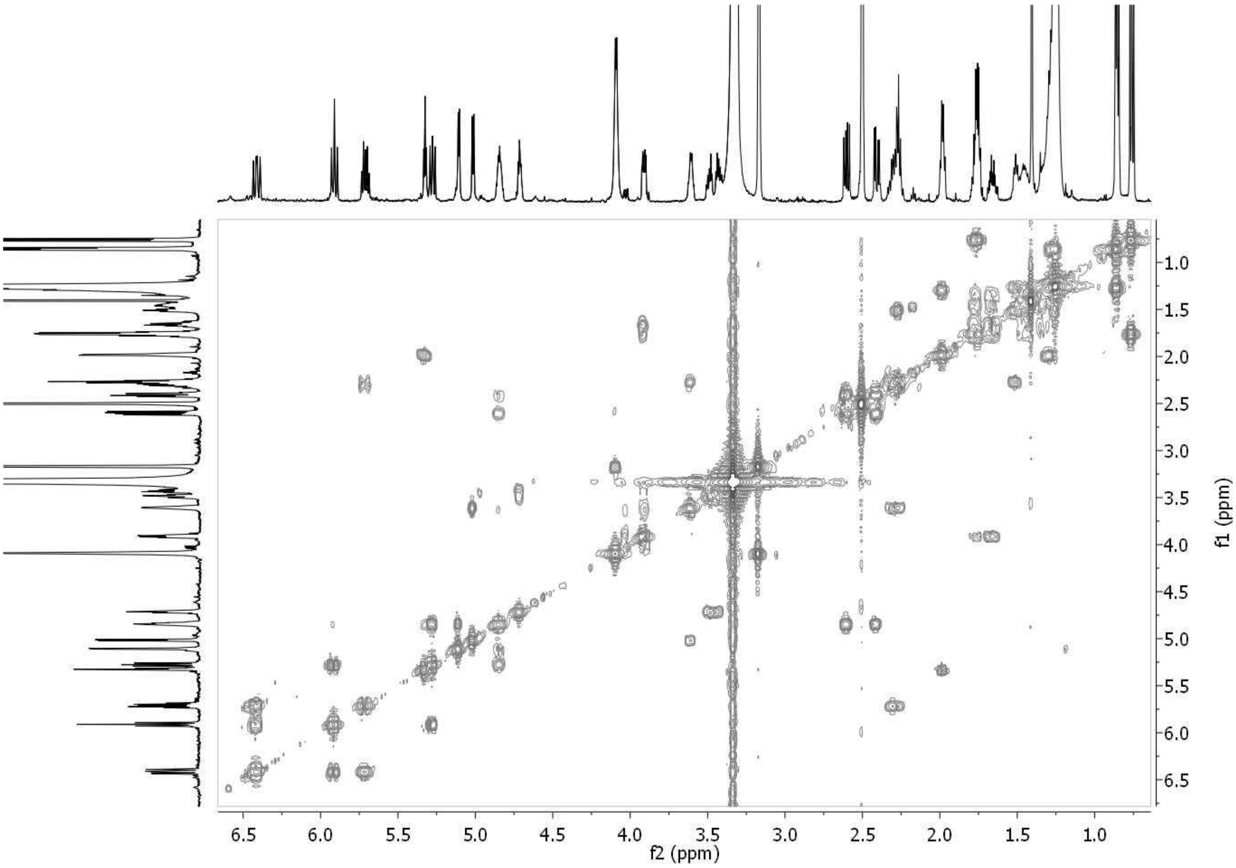

Figure S30. HSQC spectrum (600 MHz) of (**4**) in DMSO-d_6.

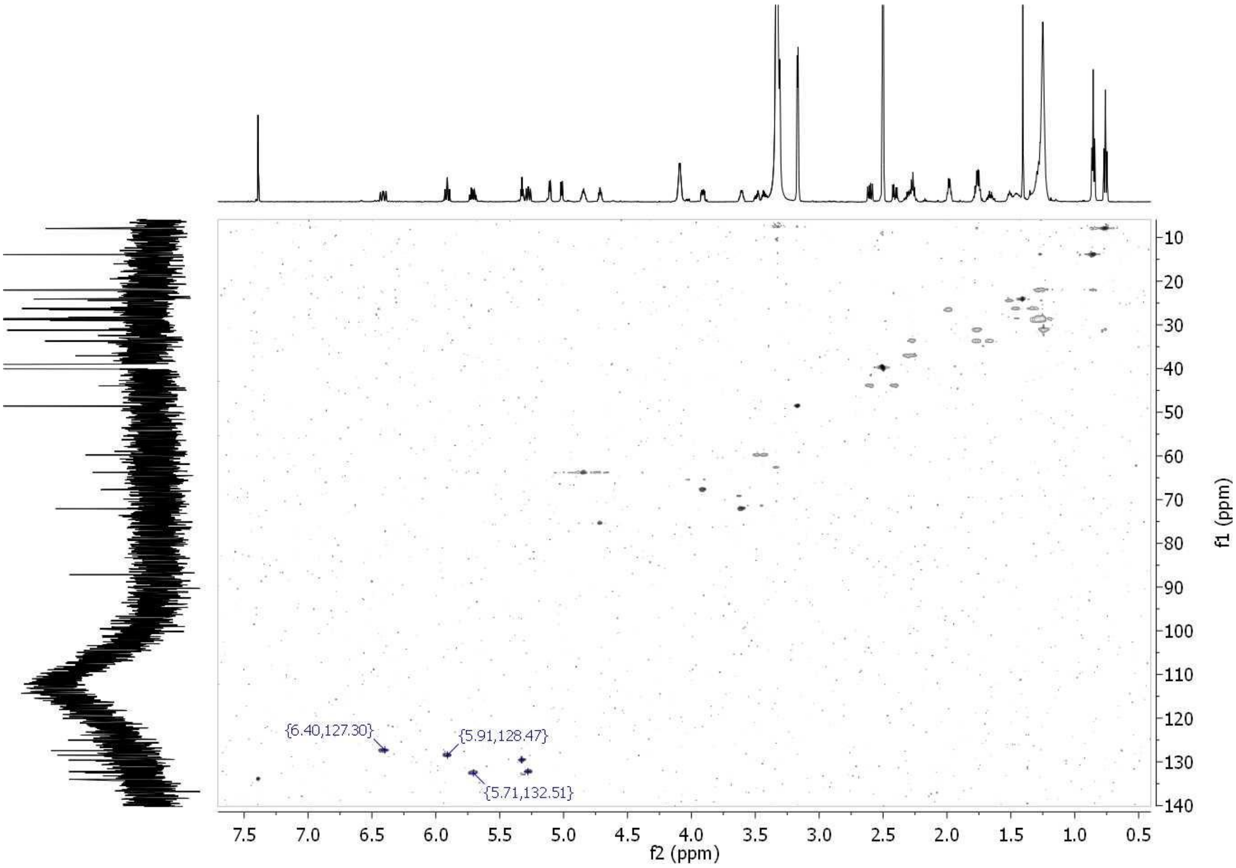

Figure S31. HMBC spectrum (600 MHz; cnst13=12) of (**4**) in DMSO-d_6.

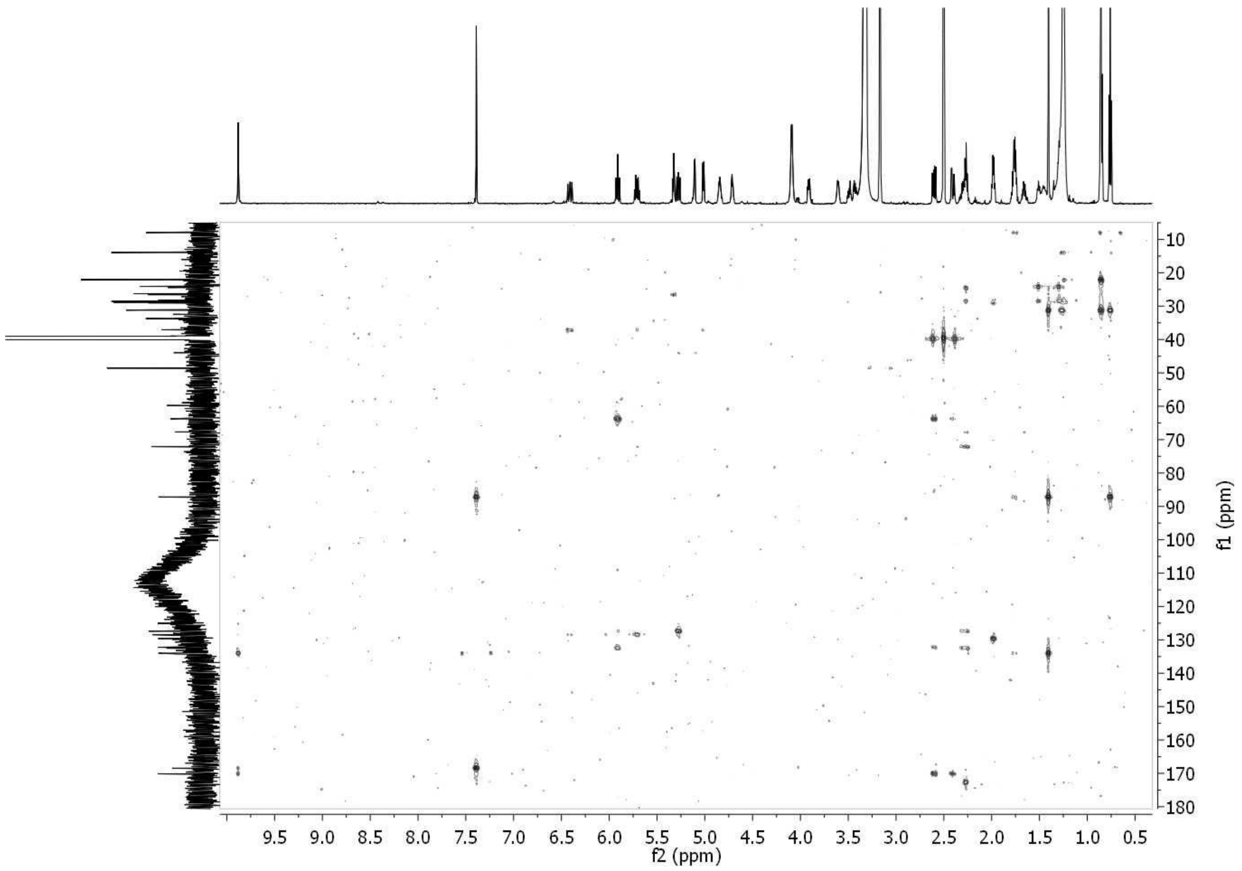

Figure S32. HMBC spectrum (600 MHz; cnst13=7.5) of (**4**) in DMSO-d_6.

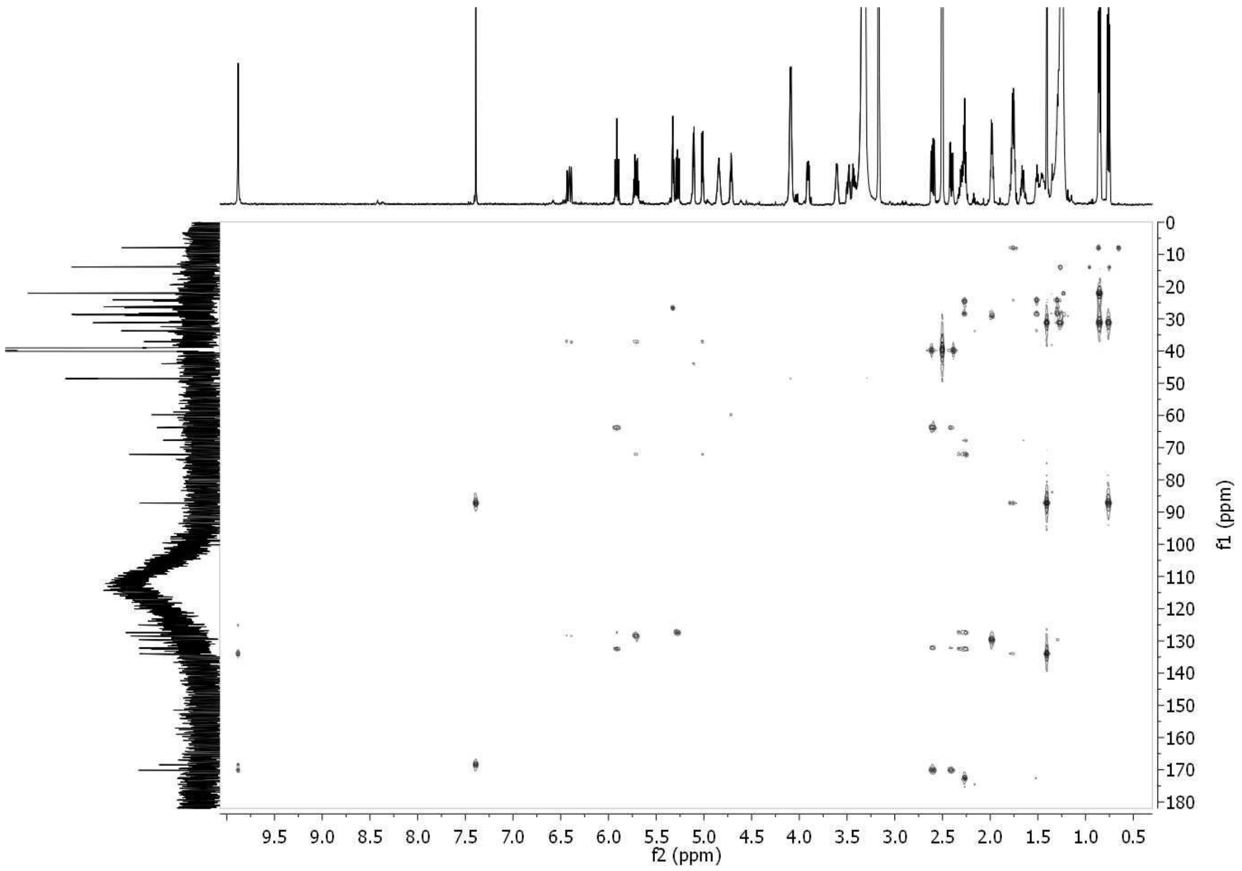

Figure S33. ^{1}H spectrum (600 MHz) of Korormicin J (**5**) in $CDCl_3$.

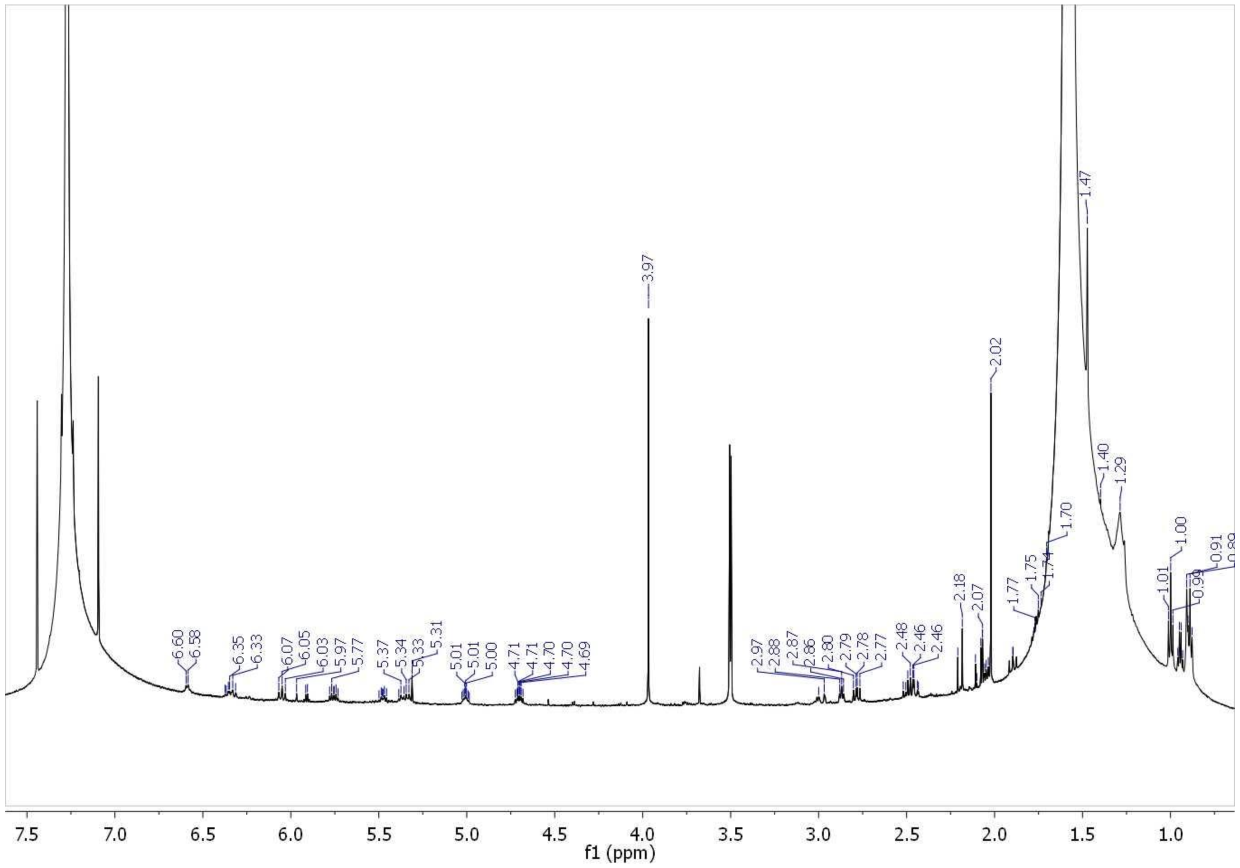

Figure S34. COSY spectrum (600 MHz) of (**5**) in $CDCl_3$.

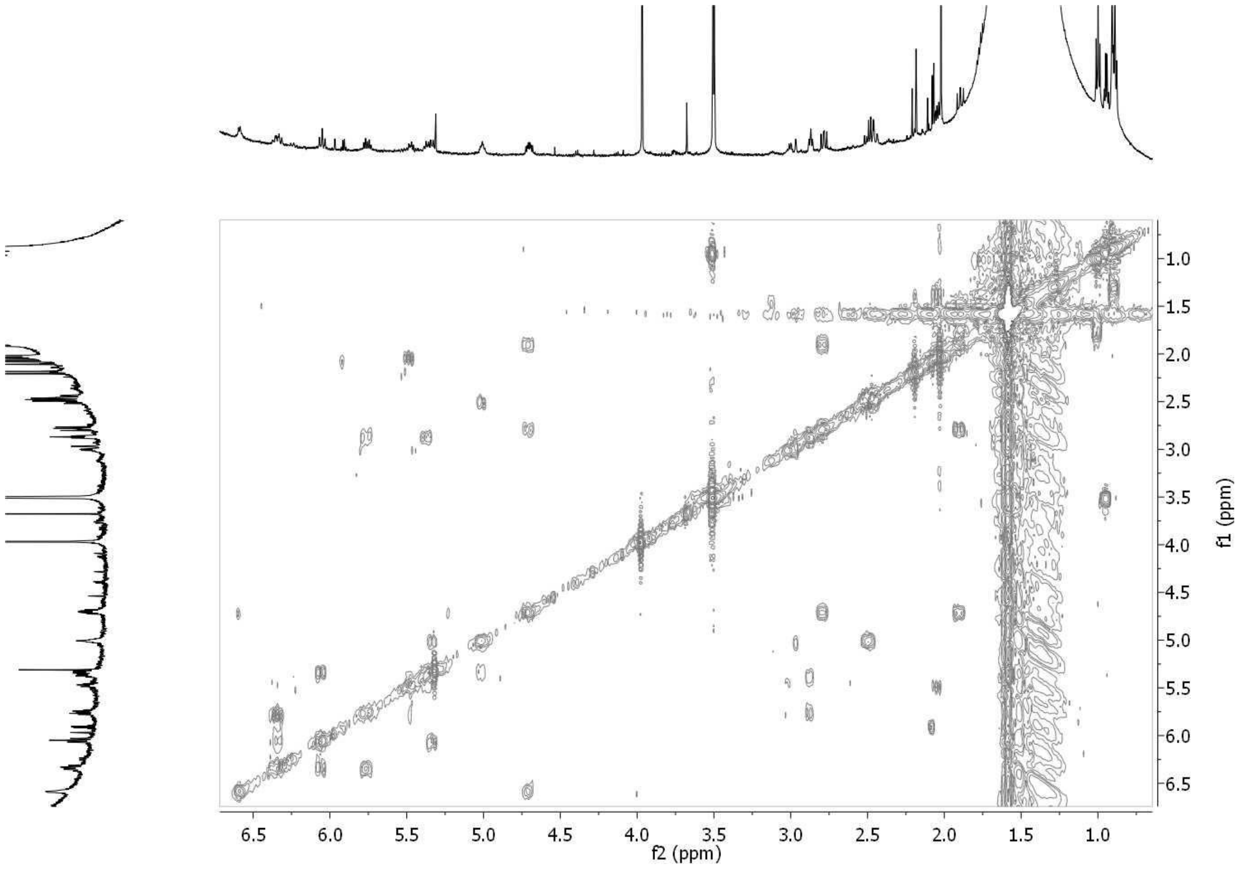

Figure S35. HSQC spectrum (600 MHz) of (**5**) in $CDCl_3$.

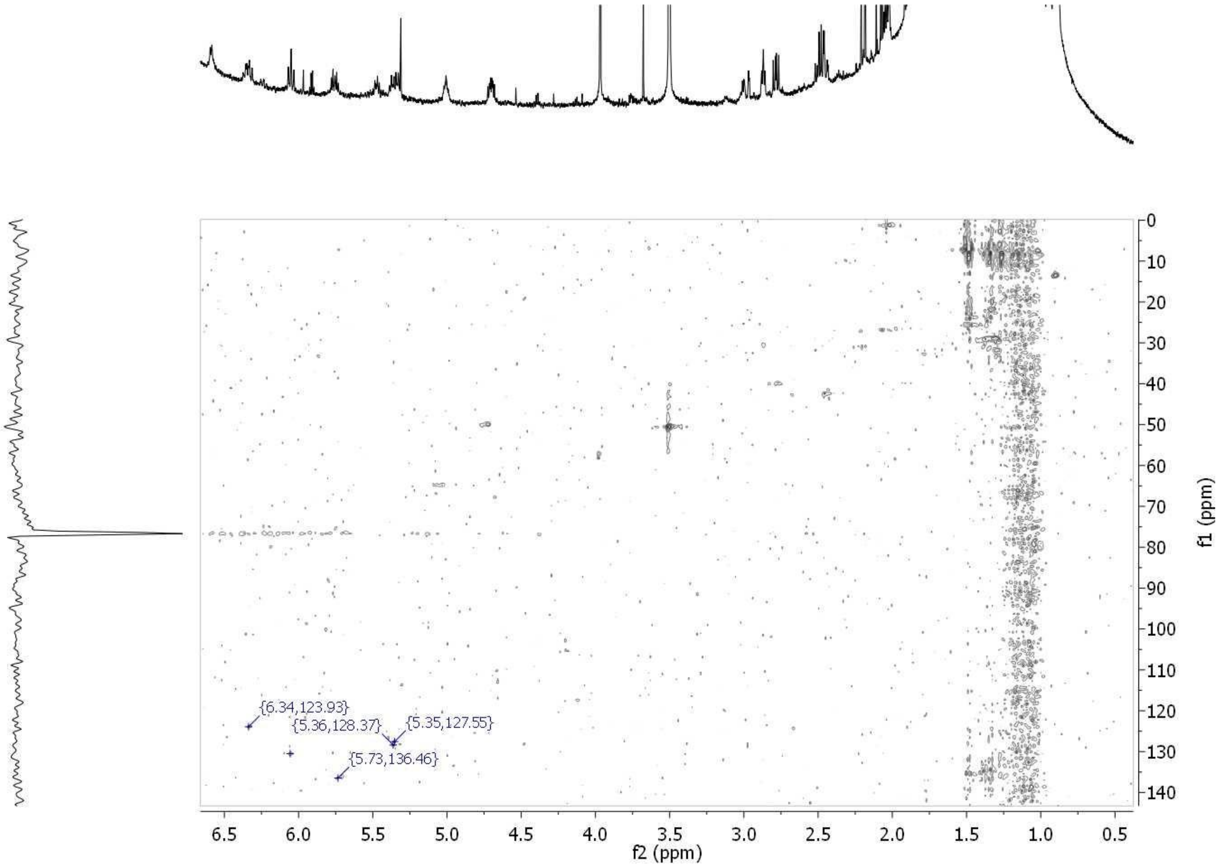

Figure S36. ^{1}H spectrum (600 MHz) of Korormicin K (**6**) in $CDCl_3$.

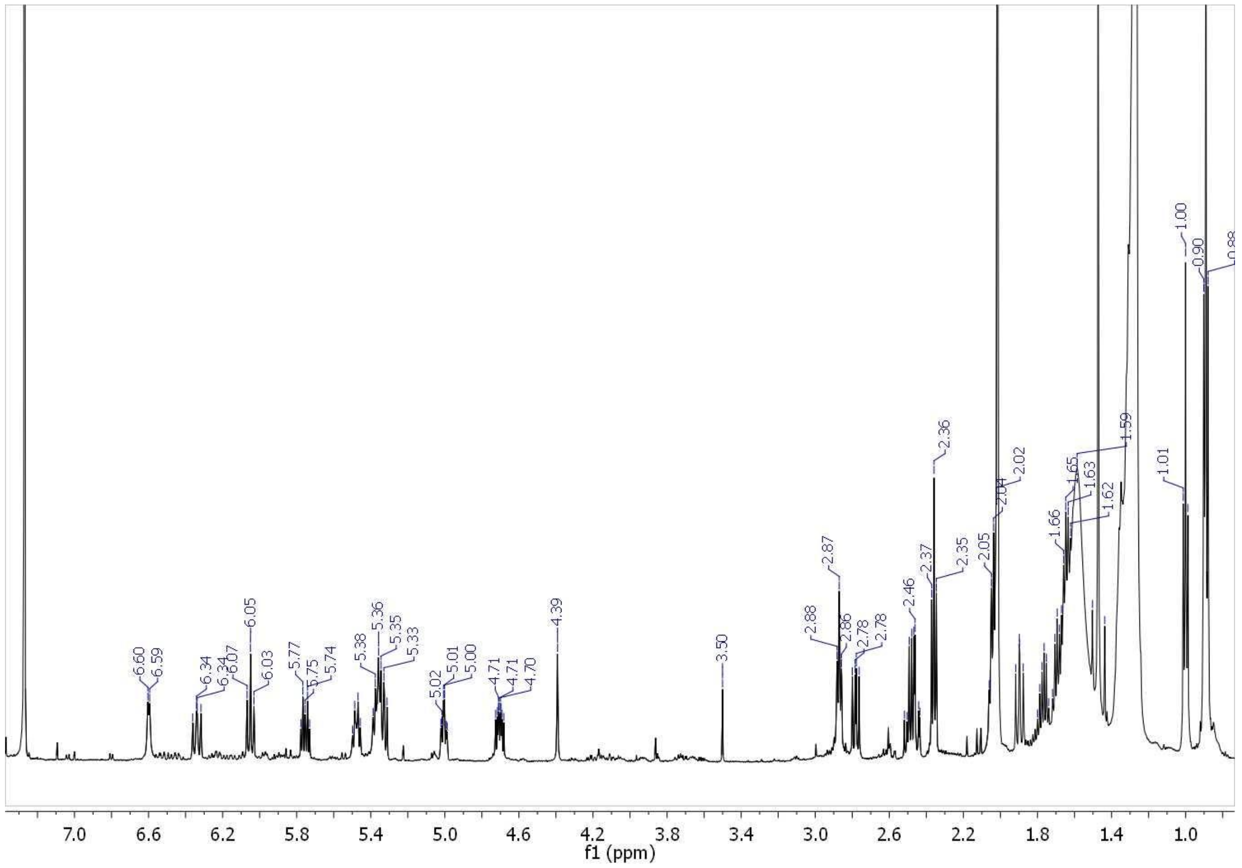

Figure S37. COSY spectrum (600 MHz) of (**6**) in $CDCl_3$.

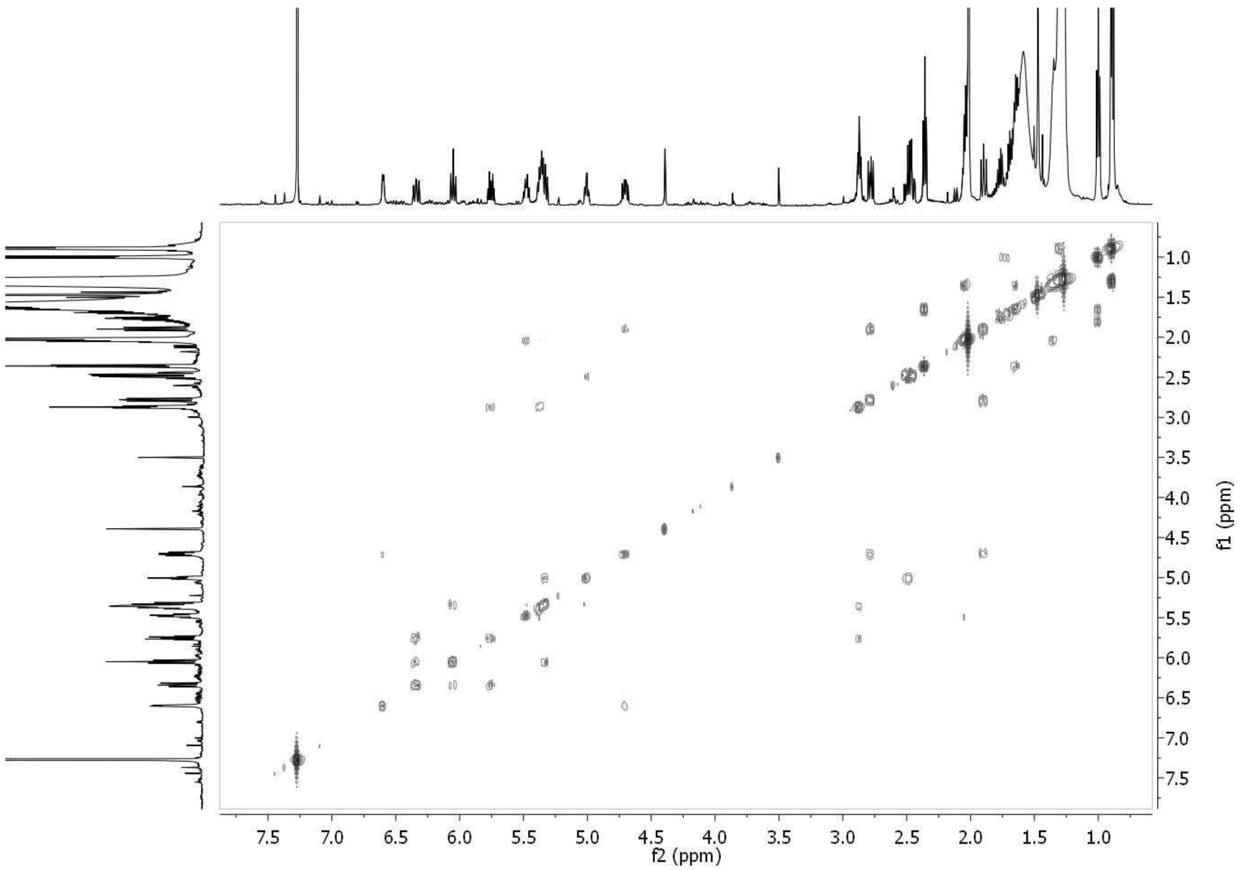

Figure S38. HSQC spectrum (600 MHz) of (**6**) in $CDCl_3$.

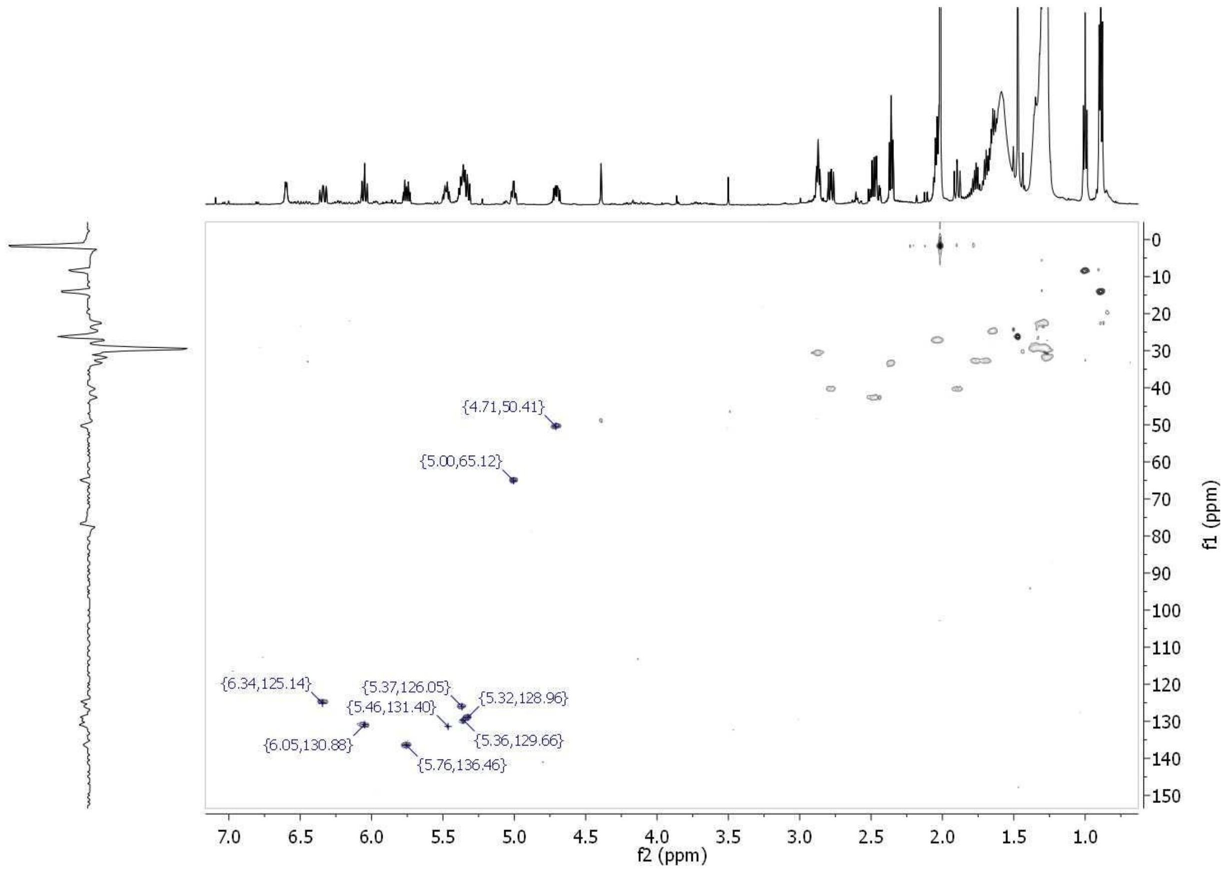

Figure S39. HMBC spectrum (600 MHz) of (**6**) in $CDCl_3$.

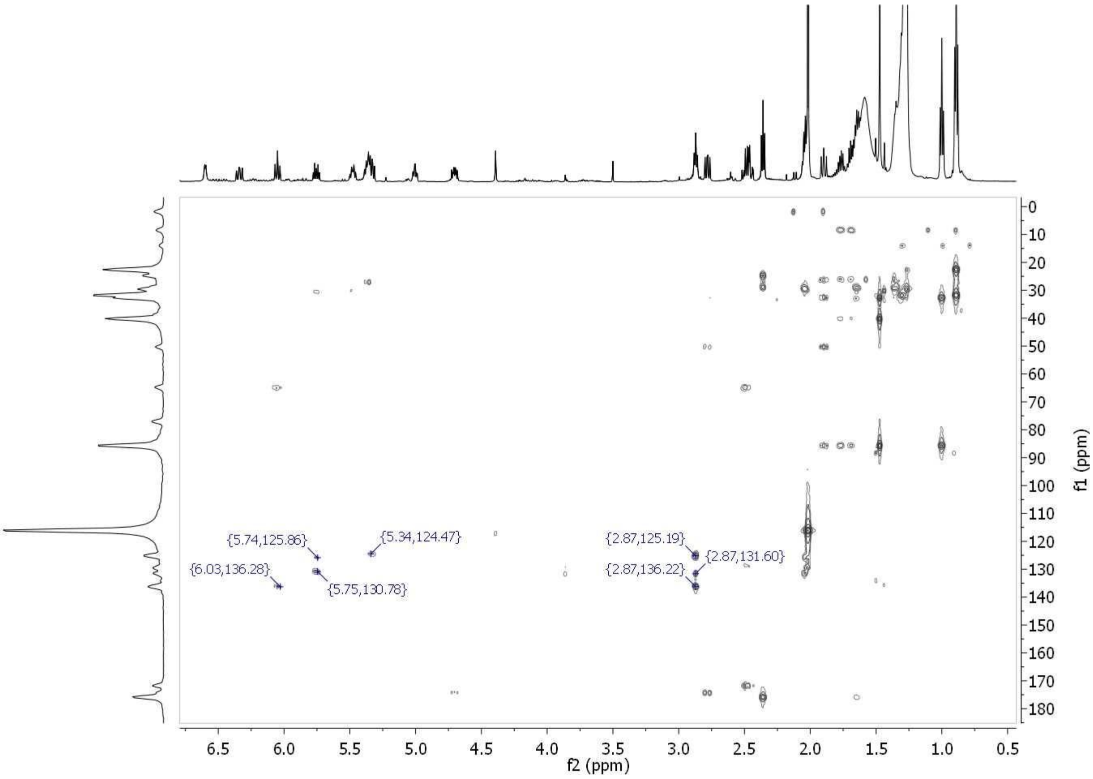

Figure S40. Selective nOe spectrum (600 MHz) of (**6**); H-3 in CDCl3.

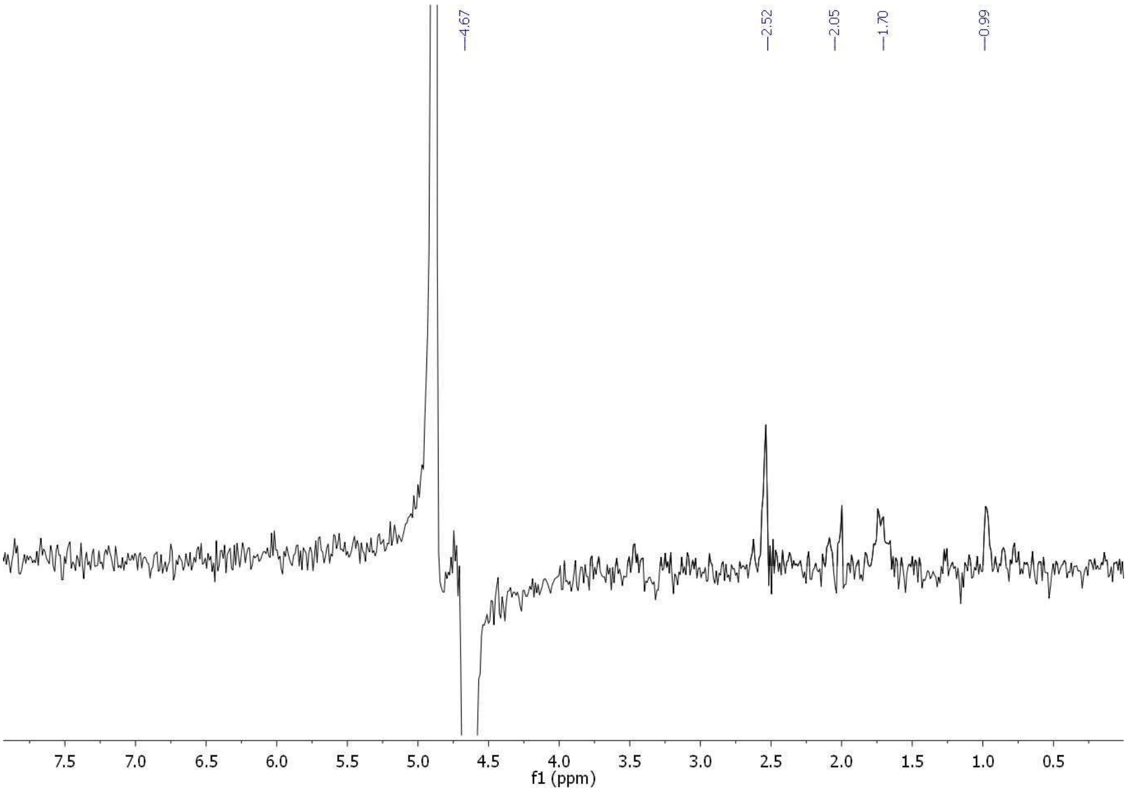

© 2014 by the authors; licensee MDPI, Basel, Switzerland. This article is an open access article distributed under the terms and conditions of the Creative Commons Attribution license (http://creativecommons.org/licenses/by/3.0/).
